# Translational potential of medicinal plants for Alzheimer’s disease: integrated evidence from molecular mechanisms to preclinical and clinical findings

**DOI:** 10.1007/s13659-026-00621-3

**Published:** 2026-07-06

**Authors:** Shirsendu Nag Saikat, Nawfal Hasan Siam

**Affiliations:** 1 Institute of Innovation, Science and Sustainability, International Institute of Business and Information Technology, Federation University, Sydney, NSW 2000 Australia; 2 School of Pharmacy and Public Health, Department of Pharmacy, Independent University, Bangladesh (IUB), Bashundhara, Dhaka, 1229 Bangladesh

**Keywords:** Medicinal plants, Alzheimer’s disease, Neuroprotection, Phytochemicals, Cognitive impairment, Amyloid and tau pathology

## Abstract

Alzheimer’s disease (AD) is a progressive neurodegenerative disorder driven by amyloid-β accumulation, tau hyperphosphorylation, oxidative stress, neuroinflammation, mitochondrial dysfunction, and cholinergic deficits. Despite extensive research, current FDA-approved therapies provide only modest symptomatic relief, underscoring the urgent need for safe and multi-targeted alternatives. This comprehensive review synthesizes evidence from in vitro, in vivo, and clinical studies on 15 medicinal plants and their 21 active compounds with therapeutic relevance to AD. Studies were included based on defined screening criteria: reported neuroprotective activity in at least one AD-related pathway, mechanistic evidence from preclinical or clinical investigations, and publication between 2015 and 2025. Analysis demonstrates that numerous plant extracts and isolated compounds including those from *Allium* *sativum*, *Bacopa* *monnieri,* *Centella* *asiatica,* *Crocus* *sativus,* *Curcuma* *longa,* *Ginkgo* *biloba,* *Hericium* *erinaceus,* *Melissa* *officinalis,* *Nigella* *sativa,* *Salvia* *miltiorrhiza,* *Vitis* *vinifera,* and others exert neuroprotective actions through convergent mechanisms. These include attenuation of oxidative and inflammatory pathways, inhibition of acetylcholinesterase and BACE-1, suppression of amyloid and tau pathology, enhancement of synaptic plasticity, mitochondrial support, and activation of neurotrophic signaling such as BDNF, TrkB and NRF2. Several compounds, including crocin, crocetin, curcumin, bilobalide, tanshinone IIA, bacosides, thymoquinone, and salvianolic acid B, demonstrate strong mechanistic activity across multiple AD-related pathways. Clinical trials further validate translational potential, with improvements reported in cognitive performance, behavioral symptoms, biomarker regulation, and daily functioning in individuals with mild cognitive impairment, AD, or age-related cognitive decline. Together, the evidence highlights medicinal plants as promising complementary or alternative strategies for modifying disease-relevant mechanisms and supporting cognitive health. Continued high-quality clinical investigations and optimized formulations are essential to advance these candidates toward therapeutic application.

**Graphical abstract:**

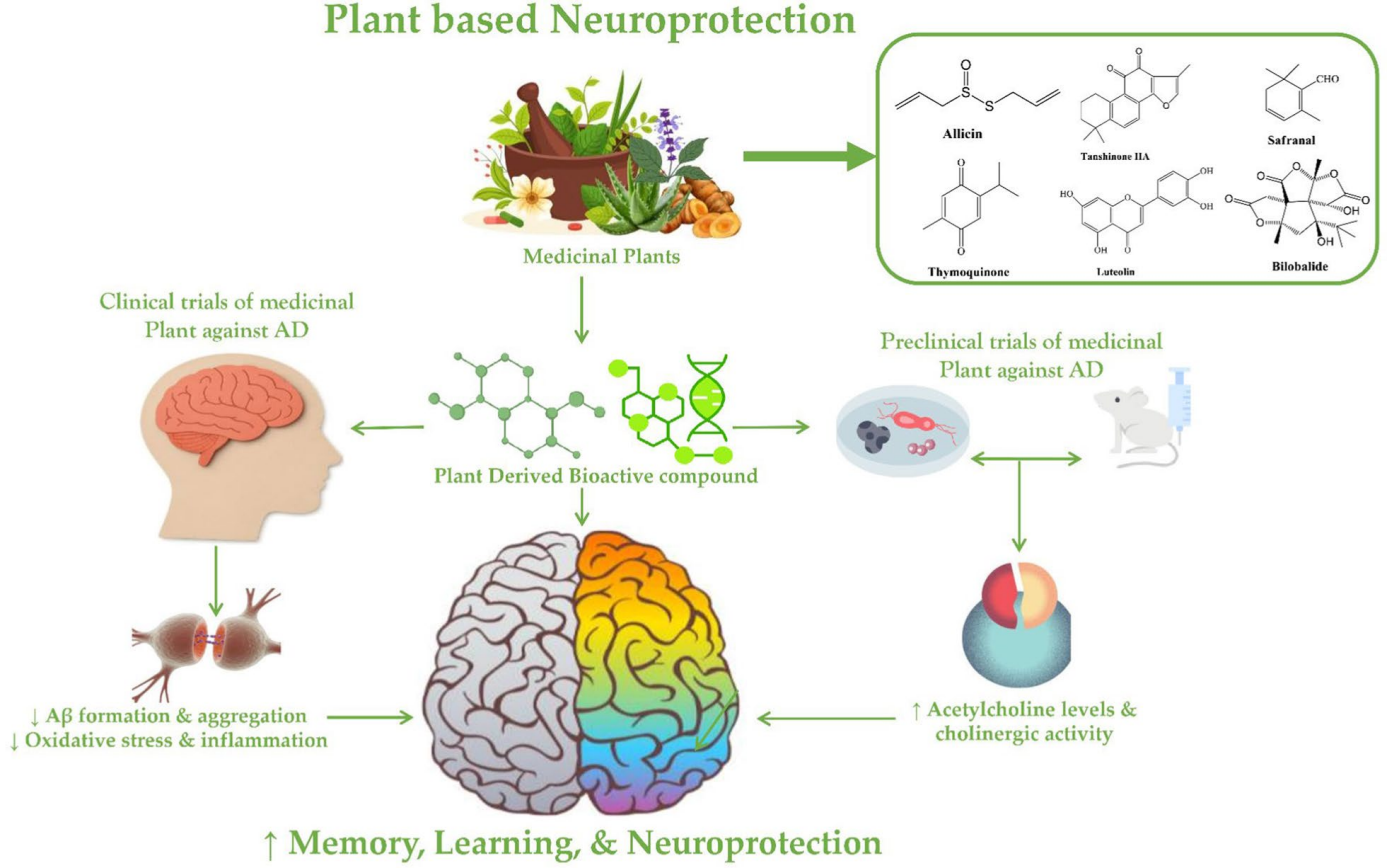

## Introduction

Alzheimer’s disease (AD) is biologically characterized by extracellular *β-*amyloid (A*β*) plaque deposition and intracellular neurofibrillary tangles composed of hyperphosphorylated tau, which together drive a cascade of progressive neurodegeneration. Clinically, AD manifests in both genetic and sporadic forms and typically presents with amnestic impairments, although several less common variants demonstrate prominent non-amnestic cognitive decline [1, 2]. A broad set of pathological disturbances accompany these hallmark lesions, including mitochondrial dysfunction, cerebral hypometabolism, oxidative stress, neuroinflammation, and disruption of the blood–brain barrier. Early structural damage typically follows the Papez circuit affecting regions such as the hippocampal formation, entorhinal cortex, anterior thalamic nuclei, and posterior cingulate cortex. As AD advances, atrophy extends across multiple cortical and subcortical areas, with the posterior cingulate cortex, precuneus, and medial orbitofrontal regions showing disproportionate vulnerability. In contrast, auditory, sensorimotor, and premotor areas remain relatively preserved. Clinically, AD manifests as a gradual decline in memory, executive functioning, visuospatial processing, language, and speech production [3, 4]. These cognitive deficits impair daily activities and are often accompanied by behavioral and psychological symptoms of dementia (BPSD), which tend to emerge as the disease progresses [5]. Although the precise initiating mechanisms are still debated, two pathological signatures remain central to AD: extracellular Aβ fibrils forming senile plaques and intracellular neurofibrillary tangles composed of hyperphosphorylated tau. Current evidence suggests that Aβ accumulation represents an early and critical event in disease pathogenesis, with fibrillar deposits appearing years often decades before visible clinical symptoms [6] The global health burden of AD continues to rise sharply. Data from the Global Burden of Disease Study place AD among the fastest-growing causes of mortality. Projections estimate that the number of individuals living with AD will increase from 26.6 million in 2006 to approximately 107 million by 2050 [7], with about 16.5 million cases expected in Europe. Importantly, nearly 68% of this increase is anticipated in low- and middle-income countries, reflecting demographic shifts and expanding life expectancy [8]. Most dementia cases occur in adults over 60 years of age. As human lifespan continues to lengthen, the number of people affected by AD is growing rapidly, driving substantial research efforts to understand disease mechanisms and identify effective treatments. Despite these advances, current therapeutic strategies remain limited and do not halt or reverse the underlying neurodegenerative process [9].

Currently approved therapies for AD provide only modest symptomatic relief and do not stop disease progression. FDA-approved acetylcholinesterase inhibitors (donepezil, galantamine, rivastigmine) and the NMDA receptor antagonist memantine improve cognition and daily functioning to a limited extent and are often associated with side effects and adherence challenges. These limitations underscore the urgent need for alternative strategies, particularly those derived from medicinal plants, which offer multi-targeted neuroprotective mechanisms and favorable safety profiles [10]. Because of these limitations, increasing attention has turned toward herbal and traditional medical systems, including Ayurveda and traditional Chinese medicine (TCM), which are valued for their natural origin, favorable safety profiles, and generally lower incidence of adverse effects compared with many modern pharmacological agents [11, 12]. Over the past decades, natural products have been widely examined for their neuroprotective potential. Crude plant extracts, isolated phytochemicals, and standardized herbal formulations have shown meaningful promise in experimental models and early clinical evaluations for managing or delaying AD pathology [13, 14]. Many of these natural compounds demonstrate mechanisms relevant to AD such as antioxidant effects, anti-inflammatory actions, AChE inhibition, modulation of amyloid and tau pathways, and protection against synaptic dysfunction supporting their potential therapeutic role. Numerous phytochemicals with neuroactive properties continue to be evaluated for AD, adding to a growing body of evidence supporting their translational relevance [15]. Historically, many modern medicines trace their origins to plant derived compounds, and large portions of early pharmacopeias were built around herbal preparations. While the rise of modern pharmacology led to a substantial decline in the clinical use of traditional remedies, interest in herbal therapeutics has re-emerged, particularly for chronic, psychiatric, and neurological disorders. Several factors contribute to this trend: dissatisfaction with the limited effectiveness of conventional drugs, the appeal of treatments aligned with cultural or personal beliefs, and a growing preference for greater autonomy in healthcare decisions [16]. Importantly, many plant-derived constituents, including alkaloids, flavonoids, terpenoids, and diverse phenolic compounds, have demonstrated activity on key brain receptors and enzymes. A significant number exhibit robust AChE inhibitory activity, suggesting that herbal medicines may hold meaningful value in neurological disease management [17]. Both preclinical and clinical studies further support the scientific basis for using medicinal plants in treating cognitive disorders, reinforcing their potential as complementary or alternative options for AD therapy [16]. The aim of this study is to comprehensively evaluate the therapeutic potential of medicinal plant and their bioactive phytoconstituents in the prevention and management of AD. Specifically, the study seeks to explore their neuroprotective mechanisms such as antioxidant activity, anti-inflammatory effects, acetylcholinesterase inhibition, and modulation of amyloid-*β* and tau pathology through evidence derived from in vitro, in vivo, and clinical research. By integrating current findings on whole plant extracts, isolated phytochemicals, and dietary applications, the study aims to highlight the translational value of medicinal plants as complementary or alternative strategies for combating AD-related cognitive decline.

## Methodology

### Literature search strategy

A comprehensive literature search was conducted using Google Scholar, PubMed, Cochrane Library, ScienceDirect, and Scopus databases. The search utilized Boolean combinations of keywords to maximize relevance, including: “Alzheimer’s disease” OR “dementia” AND (“medicinal plant” OR “plant extract” OR “phytochemicals”) AND (“memory enhancement” OR “cognitive improvement” OR “neuroprotective”) AND (“in vitro” OR “in vivo” OR “preclinical” OR “clinical trials”). Additional keywords targeting specific plants and their bioactive compounds (e.g., *Allium* *sativum*, *Bacopa* *monnieri,* *Centella* *asiatica,* *Crocus* *sativus,* *Curcuma* *longa,* *Ginkgo* *biloba*) AND (“isolated compound” OR “phytochemical”) were applied to focus on studies investigating both plant extracts and isolated active compounds. The review included studies published between 2015 and 2025, with a total of 166 relevant studies identified. of these, 66 studies were published between 2015 and 2020, while 100 studies were published between 2021 and 2025, indicating an increasing research focus on plant-based neuroprotective interventions in recent years.

The selection criteria included in vitro studies, in vivo preclinical studies using animal models of AD or cognitive impairment, and clinical trials assessing cognitive outcomes. Specific inclusion criteria encompassed studies evaluating plant extracts or isolated compounds for: memory enhancement, antioxidant effects, inhibition of cholinesterase activity, anti-neuroinflammatory effects, or other molecular mechanisms relevant to Alzheimer’s pathology. Key data extracted from each study included the year of publication, plant species, plant part used, type of extract or compound, experimental model, dosage, duration, observed outcomes, and mechanistic insights.

Following full-text screening, studies were excluded for predefined reasons: studies were excluded due to lack of direct relevance to Alzheimer’s disease or cognitive impairment, studies did not report mechanistic or efficacy-related outcomes, 28 studies focused on non-plant-derived interventions, studies were review articles, conference abstracts, or commentaries, and studies were excluded due to non-English language or insufficient methodological detail. This systematic screening process ensured transparency and methodological rigor, resulting in the inclusion of high-quality studies that formed the basis for evaluating the therapeutic potential of medicinal plants and their bioactive compounds in AD.

## Epidemiology

The global burden of AD continues to rise at an alarming pace. Recent estimates indicate that 52.56 million people aged 60 years or older were living with AD in 2021, with an age-standardized prevalence of 5146.66 cases per 100,000 population. Projections suggest that this number may triple by 2050 as the world’s population grows older. These estimates are based on data collected from 204 countries and classified into five socio-demographic index levels and 56 regions, including South and East Asia, Australasia, the Americas, and Eastern and Western Europe [18]. Variations in prevalence across these regions reflect interactions between genetic risk factors and environmental and lifestyle influences such as diet, education, physical activity, and vascular health. The interplay between genetics and lifestyle has been highlighted in recent work demonstrating that the APOE ε4 allele interacts with modifiable factors such as exercise, dietary habits, and smoking to influence AD risk [19]. Evidence from national and regional studies reveals striking demographic differences. In one region of China, AD affected 3.20% of individuals aged 60 years and above, with a markedly higher prevalence in women (4.17%) than in men (2.30%). Illiteracy emerged as an important determinant, with rates more than double those of individuals who had some level of education [20]. Across Asian populations generally, differences in education, urbanization, and cardiometabolic risk factors (e.g., hypertension, diabetes) contribute substantively to regional disparities in AD prevalence, as socioeconomic and lifestyle factors influence cognitive reserve and vascular health [21]. Data from 18 Indian studies report an overall prevalence ranging from 2 to 3.5%, though some subgroups showed rates as high as 5.1%. Women again bore a disproportionate burden (7.2% vs. 3.8% in men), with risk heightened by aging, low education, unmarried status, heavy alcohol use, cardiovascular and neurological conditions, depression, and nuclear living arrangements [22]. These findings echo broader evidence that lower education and vascular risk factors are consistently ranked among the most prominent modifiable AD risk factors (e.g., hypertension, diabetes) globally, with low education often outweighing smoking and inactivity among diverse populations [23]. In England, the prevalence of clinically diagnosed AD reached 378.39 per 100,000, compared with 292.81 per 100,000 for early-stage AD. The growing elderly population accounted for much of this increase, and prevalence was substantially higher in women. Socioeconomic deprivation also played a critical role, with the highest rates observed in the most disadvantaged communities [24]. Socioeconomic deprivation is linked with higher cardiovascular risk, reduced healthcare access, and lower lifetime cognitive stimulation factors that together accelerate AD pathology. Disparities by socioeconomic status persist even after adjusting for genetics, underscoring the role of environmental influences [25]. In the United States, some of the world’s highest AD prevalence levels have been documented, reaching 12.9% in states such as Maryland and New York, and 16.6% in Miami-Dade and the Bronx. Women had a 13% higher risk than men, while Black and Hispanic populations showed a markedly higher prevalence compared with White individuals. Lower educational attainment was also closely linked with increased risk [26]. This aligns with large cohort findings showing that racial and ethnic disparities in AD and dementia risk reflect both genetic risk, including higher APOE ε4 prevalence in some groups, and greater burdens of comorbidities like obesity, hypertension, and diabetes that modulate risk [25, 27]. Canada has experienced a steady rise in AD prevalence as well, increasing from 0.14% in 1994 to 0.80% in 2014, with a peak of 1.06% in 2013. Although women historically had higher rates, men have shown a sharper increase in recent years. Contributing factors include aging, lower education levels, diabetes, hypertension, and overweight [28]. These trends are consistent with evidence that vascular risk factors and lifestyle behaviors contribute substantially to AD risk, particularly in aging Western populations where cardiometabolic disease prevalence is rising [23]. In Australia, estimates of dementia prevalence range from 123,818 to 487,500 cases, with age- and sex-standardized rates between 21.4 and 65.9 per 1000 people aged 60 and above. These figures may underestimate the true burden, owing to misdiagnosis, late diagnosis, and limited data on institutionalized older adults particularly older women [29]. Socioeconomic and geographic disparities within Australia, including access to healthcare and preventive services, likely contribute to regional variation, while lifestyle determinants such as obesity and physical inactivity also influence prevalence [23]**.** Similarly, a New Zealand study comparing two population groups reported an age-adjusted dementia prevalence of 5.4% among non-Māori and 7.4% among Māori. The highest prevalence was seen in Māori men aged 85 years and older (27.2%). These disparities reflect the combined influence of chronic illnesses, aging, unequal access to dementia-related healthcare, and broader ethnic inequities [30]. Ethnic disparities in AD prevalence are influenced by differential exposure to modifiable risk factors, lifetime socioeconomic disadvantage, and healthcare access, which amplify risk beyond genetics alone [25]. In North Africa and the Middle East, the age-standardized AD prevalence reached 772.7 per 100,000, affecting an estimated 2.68 million people in 2021. Women were again disproportionately affected, a pattern shaped by factors such as older age, limited education, obesity, diabetes, hypertension, heart disease, smoking, sedentary lifestyles, dietary patterns, genetic susceptibility including ApoE4, and disruptions to healthcare during the COVID-19 pandemic [31]. Regional reviews highlight that rising cardiometabolic diseases, urbanization, and lifestyle changes including diet and physical inactivity are major contributors to the increasing burden of AD in the Middle East and North Africa (MENA) region [32]. Overall, Aging, gender, lack of education, socioeconomic deprivation, inactive lifestyle, disparities, chronic diseases conditions, psychosocial factors, and healthcare system limitations like misdiagnosis and a lack of access to healthcare are the most common factors impacting the prevalence of Alzheimer's disease across all regions. These findings demonstrated the importance of broad, focused public health strategies across all regions [18, 20, 22, 24, 29–31].

## Therapeutic applications

### Medicinal plant and their benefits for AD

**Fig. 1:**
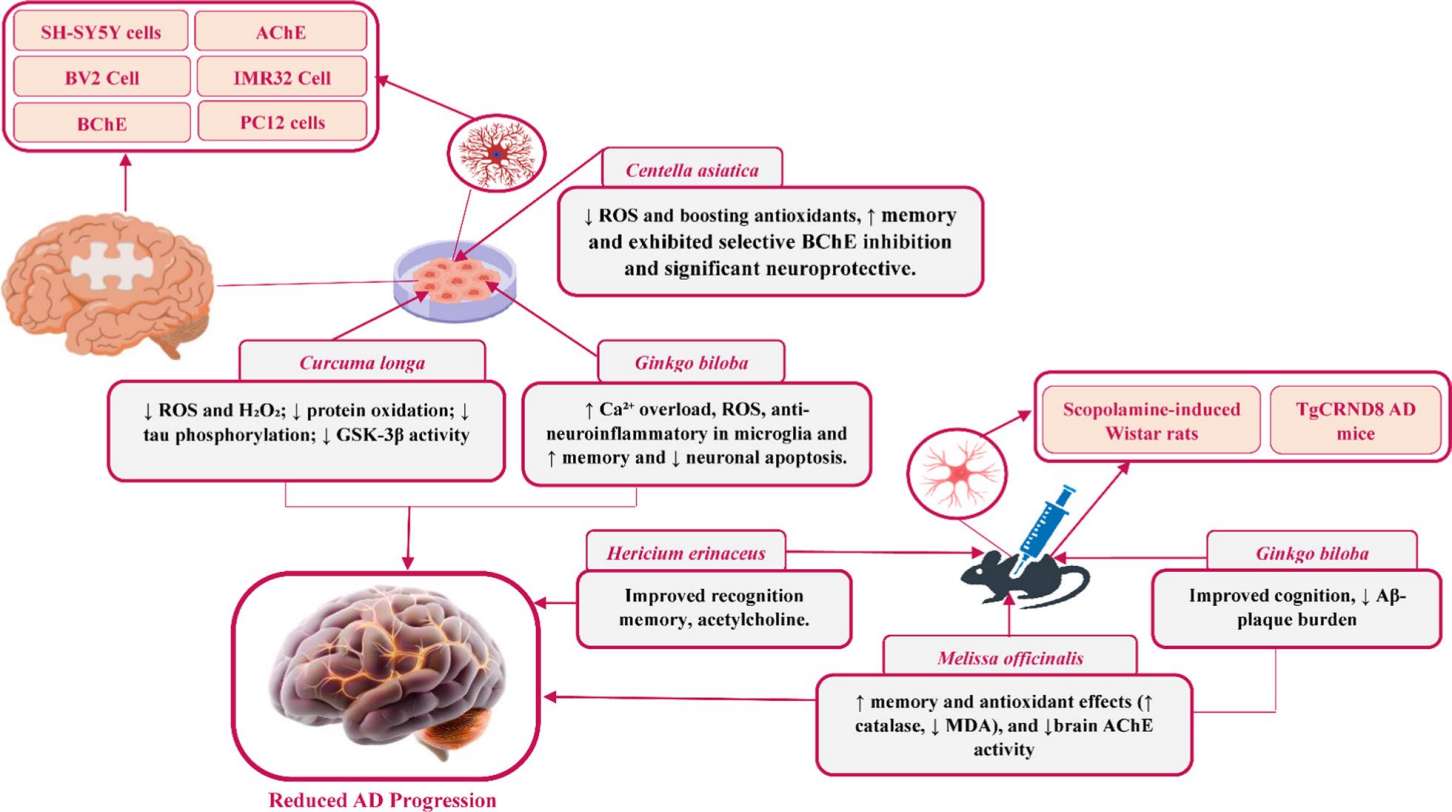
Proposed in vivo and in vitro mechanisms of action of *Medicinal* *Plant* in Alzheimer. (↑) indicates increased; (↓) indicates reduced; ROS, reactive oxygen species; GSK-3β, glycogen synthase kinase-3 beta; AChE, acetylcholinesterase; BChE, butyrylcholinesterase

The integration of medicinal plants into diets offers significant potential for improving cognitive health, particularly in preventing and managing AD. Table 1 highlights a range of plant, their therapeutic benefits, and modes of consumption, illustrating their profound effects on brain health. Medicinal Plant, in particular, have been recognized as a valuable source of anti-AD effects, including the reduction of specific enzyme activity, oxidative stress, Tau and A*β* protein aggregation, and neuroinflammation, as well as enhancing cellular protection and repair mechanisms (Fig. 1).

**Table 1:** Medicinal plant beneficial for AD

Plant name	Common name	Part	Extract	Study type	Dose	Pharmacological activity	References
*Allium* *sativum*	Garlic	Clove	Essential oil (hydrodistilled)	In vitro	not reported	Exhibited moderate anti-cholinesterase activity, inhibiting AChE (65.4%) and BChE (31.5%), increase memory	[33]
				In vivo, Aβ_(25–35)_-induced	1, 2, and 3% wt/wt (p.o.)		
		Clove	Essential oil	In Vivo, Male C57BL/6 mice	10 ml/kg	Increase brain-derived neurotrophic factor (BDNF) levels and decrease acetylcholinesterase (AChE) activity	[34]
*Allium* *hookeri* Thwaites	Hooker chives and garlic chives	Leaf and root	Ethanol	In vivo, scopolamine-induced C57BL/6 mice	150 and 300 mg/kg	Increase cognition via cholinergic enhancement, anti-inflammatory action, and neuroprotection	[35]
*Bacopa* *monnieri* (L.) Wettst	brahmi, water hyssop	Aerial parts	Ethanol	In vivo, Aβ_42_-injected hippocampus mode	0.25, 2, 12.5, 25, 50, 100, 200 and 400 μM	Increase memory, suppresses oxidative stress and inflammation, decrease cholinesterase, tau, and Aβ, and regulates GSK-3β, Wnt/β-catenin signaling	[36]
		Not reported	Crude extract	In vivo, colchicine-induced dementia, inflammation model	50 mg/kg	Increase memory, suppresses oxidative stress and inflammation, and decrease BACE-1-mediated Aβ formation	[37]
		Herbal powder	Aqueous	In vitro*,* IMR-32 human neuroblastoma cells treated with Aβ_42_	100 μg/mL	Decrease Aβ_42_-induced apoptosis and ROS, restored dysregulated proteins, and inflammatory and cytoskeletal pathway disturbances	[38]
*Boswellia* *sacra* Flück	Boswellia carteri the frankincense tree, the olibanum tree	Not reported	Methanol	In vivo, AlCl_3_-induced	68.75, 137.5 mg/kg	Increase cholinergic function, lowered inflammation, and reduced Aβ pathology	[39]
*Centella* *asiatica* (L.) Urb	Gotu kola and pennywort	Leaves and stems	Water	In vivo,aged transgenic AD mice (Tg2576; also, WT)	200 and 1000 mg/kg	Increase memory, mitochondrial function, and hippocampal metabolism while decrease oxidative stress	[40]
		Whole dried plant	Ethanol	In vitro, neuronal cell lines (PC12, IMR32)	25, 50, 100 µg/mL	Protects neurons by decrease ROS and boosting antioxidants	[41]
		Dried aerial	Water	In vivo, AD model (5XFAD mice)	200, 500, 1000 mg/kg/day	Increase memory, boosted NRF2 antioxidant signaling, and increase oxidative stress	[42]
*Curcuma* *longa* L	Turmeric	Rhizome	Ethanol	In vivo	100, 300 mg/kg	Blocks TLR4/NF-κB, protecting neurons and increase memory	[43]
				In vitro*,* primary rat hippocampal neurons	3–30 µg/mL		
		Rhizome	Methanol	In vitro*,* anti-amyloid fibrillation assay	0.55 µg/mL	Prevents amyloid aggregation	[44]
		Rhizome	Ethanol	In vitro*,* C6 glioma + BV2 microglia	100 ng/ml	Increase memory and increase oxidative stress and inflammation, showing neuroprotective	[45]
				In vivo, scopolamine-induced amnesia model in mice	50, 100, or 200 mg/kg		
*Crocus* *sativus* L	Saffron crocus	Not reported	Hydroalcoholic	In vivo*,* transgenic AD mouse: 5XFAD	50 mg/kg/day	Increase BBB, promoted Aβ clearance, preserved synapses, and decrease neuroinflammation	[46]
		Saffron	Hydroalcoholic	In vitro**,** P-gp induction, NLRP3 inflammasome inhibition, neuroprotection in SH-SY5Y cells	6.25–100 µg/mL	Promotes Aβ clearance, suppresses NLRP3, IL-1β, protects neurons, and increase cognition	[47]
				In vivo**,** STZ-induced dementia in rats; scopolamine-induced amnesia in mice	50 and 100 mg/kg		
		Saffron	Hydroalcoholic	In vivo, chronic scopolamine-induced cognitive impairment	50 mg/kg/day	Increase Aβ pathological manifestations	[48]
*Ginkgo* *biloba* L	Ginkgo or the maidenhair tree	Leaves	Standardised leaf extract (egb 761)	In vivo, transgenic mouse model (TgCRND8 AD mice)	240 mg/day	Increase memory, prevented synaptic protein loss, and exhibited anti-inflammatory effects	[49]
		Leaves	Standardised extract (egb)	In vivo*,* APP/PS1 transgenic mouse model	100 mg/kg	Improves cognition	[50]
		Leaves	Not reported	In vitro, human neuroblastoma SH-SY5Y cells,	25 µg/mL	Increase Ca^2^⁺ overload, ROS, and apoptosis, protecting neurons in AD models	[51]
*Hericium* *erinaceus*	Lion’s mane	Mycelium	Aqueous	In vitro, PC12 cells	50 & 100 µg/mL	Protects neurons and increase motor, cognitive, and cholinergic function	[52]
				In vivo, AlCl_3_ + d-galactose-induced	0.3 g/kg, 1 g/kg, 3 g/kg		
		Not reported	Ethanol	In vivo*,* zebrafish, scopolamine-induced memory impairment	0.5, 1, 3 mg/L	Increase memory via AChE inhibition and antioxidant protection	[53]
		Biomass (whole mushroom)	Aqueous	In vivo	200 mg/kg	Increase stress-response proteins and LXA4, limiting neuroinflammation in AD-related brain regions	[54]
*Huperzia* *Serrata* (Thunb.) Trevis	Firmoss	Whole plant dried	Aqueous and ethanol	In vivo, scopolamine-induced cognitive impairment in mice (Y-maze & passive avoidance)	30 mg/kg/day	Increase cognition by suppressing AChE activity	[55]
		Not reported	Not reported	In vitro, SH-SY5Y cells	Not reported	Protects neurons and increase memory via antioxidants and cholinergic modulation	[56]
				In vivo, scopolamine-induced mice (Y-maze & Morris water maze)			
*Melissa* *officinalis* L	Lemon balm	Leaves	Ethanol	In vivo, scopolamine-treated rats (Morris water maze)	50–400 mg/kg	Increase memory via cholinergic modulation	[57]
		Leaves	Ethanol	In vivo, rats with scopolamine-induced memory impairment	200 mg/kg	Improves memory by downregulating AChE and BACE1	[58]
		Not reported	Hydro-alcoholic extract	In vivo, intracerebroventricular Aβ_1–42_ rat model of AD	50 and 100 mg/kg	Improved passive-avoidance memory retrieval	[59]
*Nigella* *sativa* L	Black seed	Seeds	Hydro-alcoholic seed extract	In vivo, Wistar rats, ovariectomised + AlCl_3_-induced AD model	100, 200, 400 mg/kg	Improves memory and decrease Aβ, GSK3β, and NFTs	[60]
		Seed	Seed oil	In vivo	0.5, 1.0, 2.0 ml/kg	Decrease inflammation and glial activation, increase cognition	[61]
		Seeds	Methanol	In vivo	10 mg/kg	Suppresses inflammation, restores synapses, and increases spatial memory	[62]
*Petiveria* alliacea L	Guinea hen weed, or garlic weed	Leaves	Methanol	In vivo, scopolamine-induced	500 or 900 mg/kg	Protects neurons and increase memory via antioxidant and cholinergic modulation	[52]
*Salvia* *miltiorrhiza* Bunge	Red sage or Danshen	Root	Ethanol	In vitro*,* NG108-15 cells	400, 600, 1200 mg/kg	Decrease Aβ25–35-induced memory deficits and oxidative stress	[63]
				In vivo, Aβ_(25–35)_-induced mouse model	2.0 µg/mL		
		Dried root and rhizome	Water	In vitro, Aβ aggregation assay (Thioflavin T)	100 µg/ml	Blocks Aβ aggregation, decreases ROS, and protects against Aβ-induced toxicity	[64]
				In vivo, Caenorhabditis elegans AD model (GMC101 strain expressing human Aβ_1–42_)	1–4 mg/mL		
		Root/rhizome	Water	In vivo, chronic cerebral hypoperfusion model	200 mg/kg/day	Prevents BCCAo-induced brain damage via anti-inflammatory and myelin-protective mechanisms	[65]
*Vitis* *vinifera* *L*	Grap multi-target effects e	Leaves	Petroleum ether	In vivo, AlCl_3_-induced Alzheimer’s model in rats	100 mg/kg	Improves cognition, antioxidant, and anti-inflammatory mechanisms	[66]
		Seed	Seed extract	In vivo, AlCl_3_-induced Alzheimer’s model in rats	250 mg/kg and 500 mg/kg	Increase learning and memory, decrease APP, Tau expression, decrease oxidative stress and inflammation, and increase cholinergic function	[67]
		Leaves	Ethanol	In vitro*,* HT22 hippocampal neuronal cells	50 µg/m	Protects neurons from glutamate-induced oxidative stress via antioxidant gene upregulation	[68]

### Bioactive phytoconstituents of medicinal plant

**Fig. 2:**
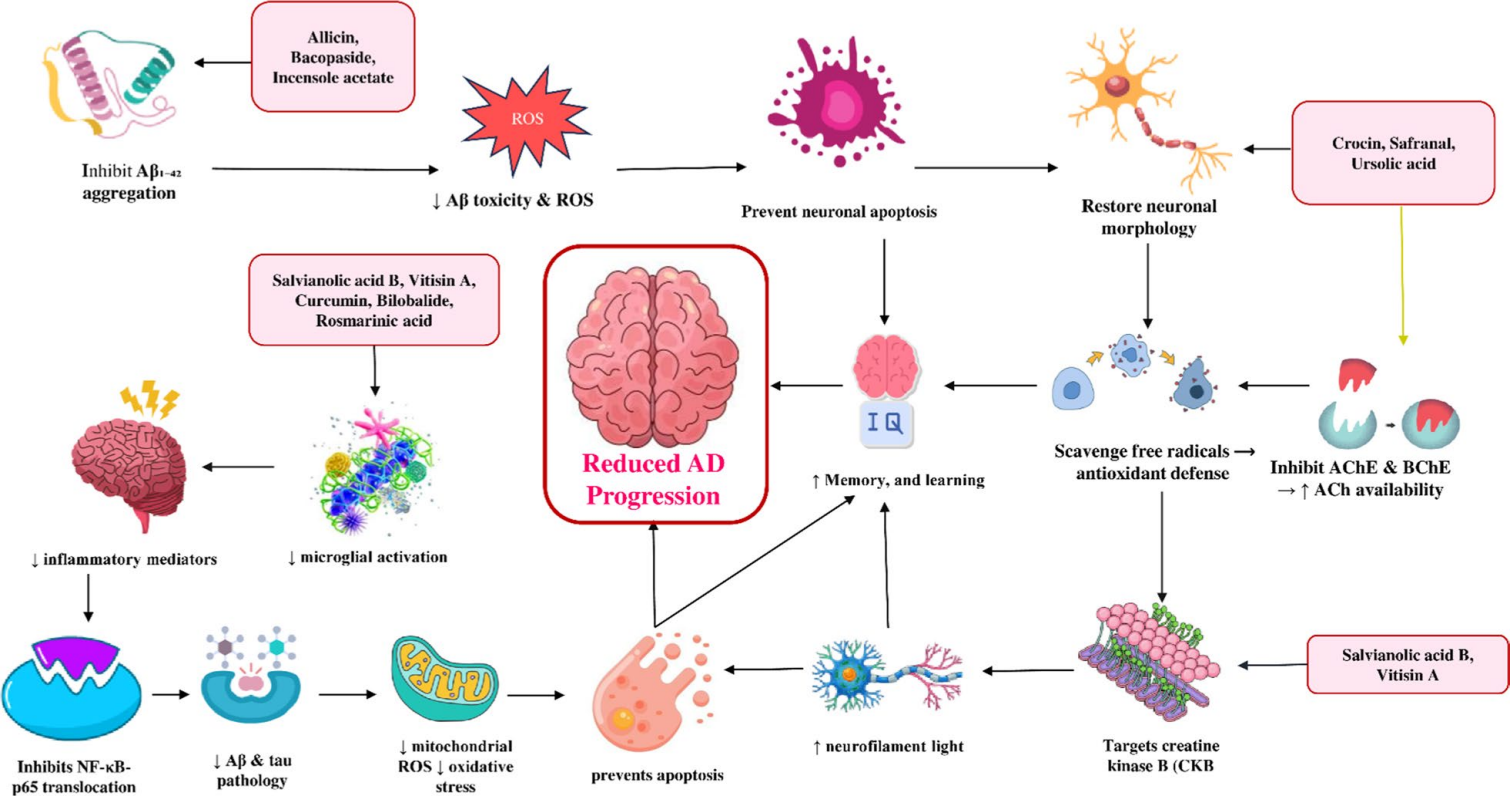
Schematic diagram illustrating the anti-Alzheimer’s mechanisms of action of various phytoconstituents found in medicinal plant. (↑) indicates increased; (↓) indicates reduced; ROS, reactive oxygen species; Nuclear factor kappa-light-chain-enhancer of activated B cells (NF-κB); Creatine Kinase B (CKB); AChE, acetylcholinesterase; BChE, butyrylcholinesterase

**Fig. 3:**
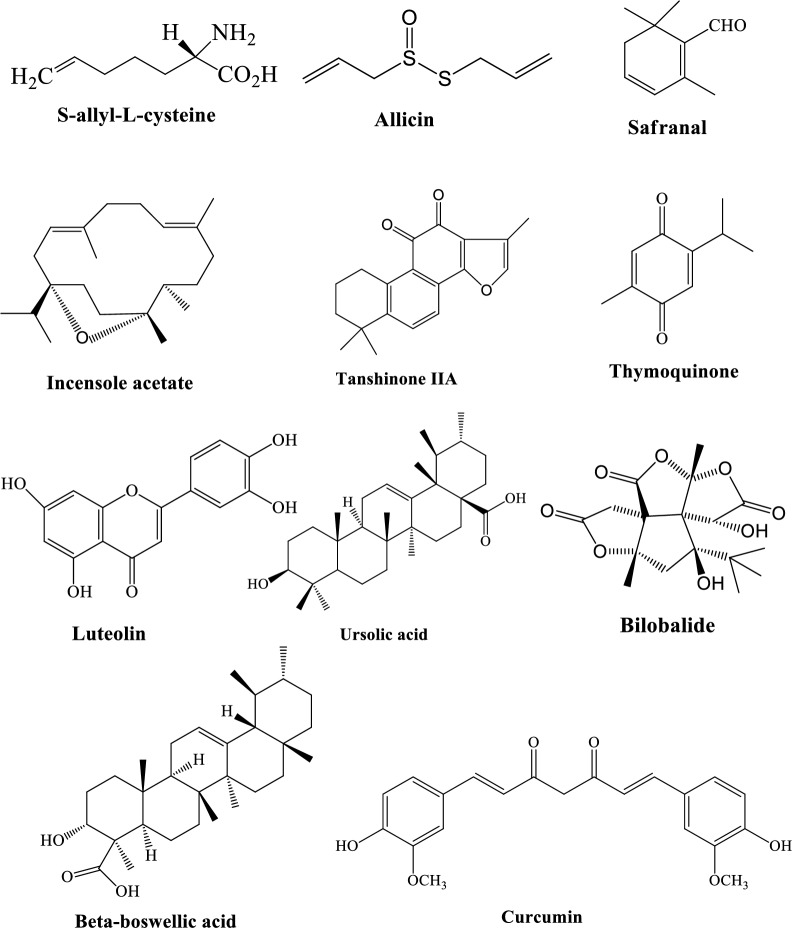

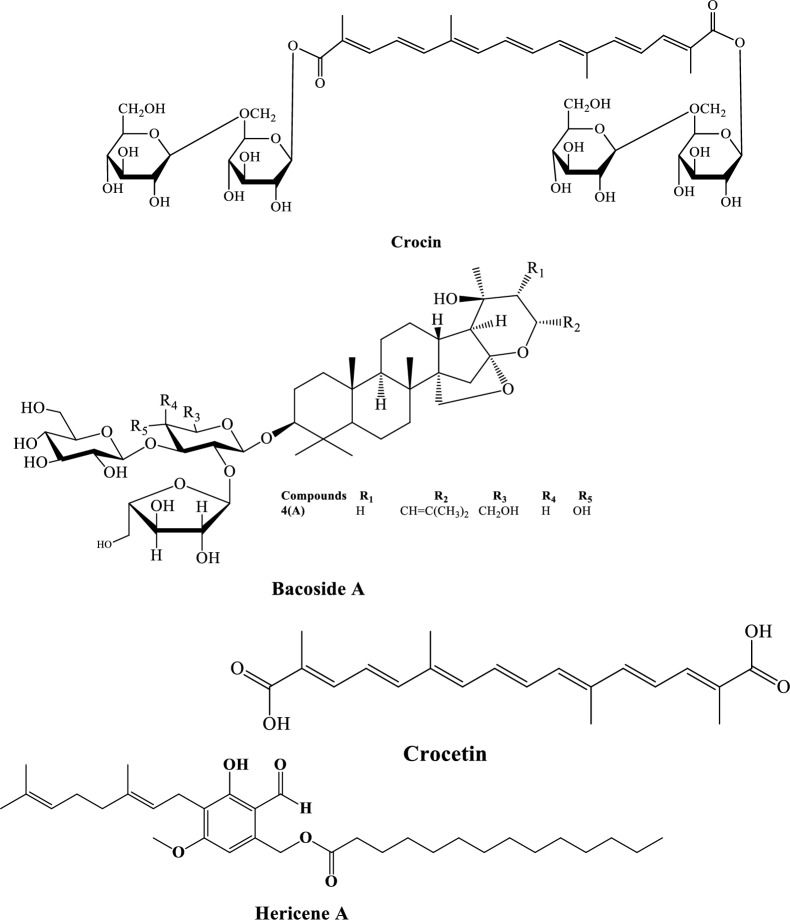

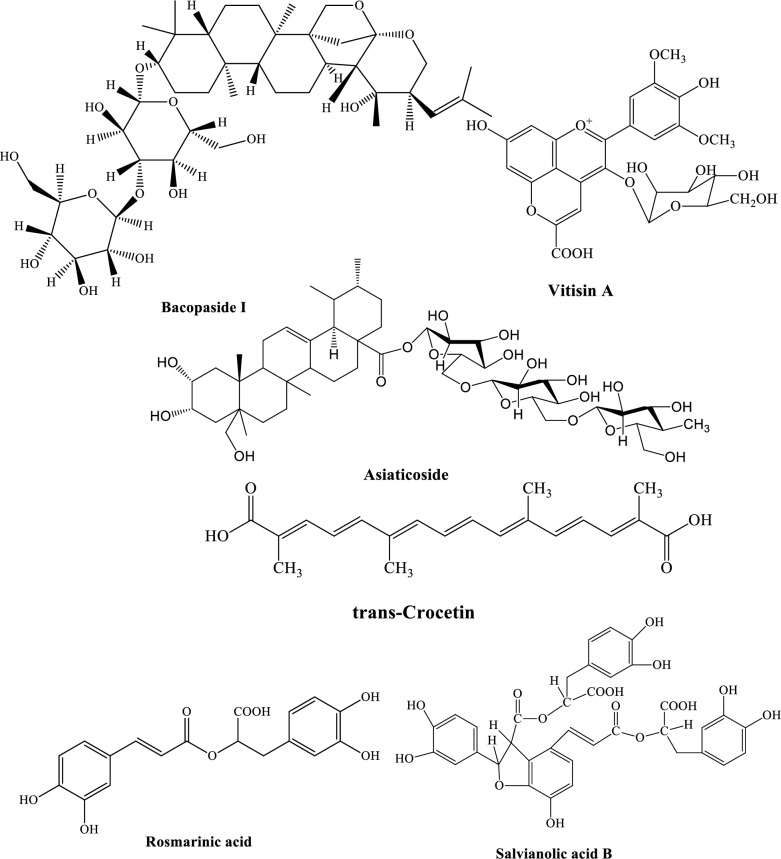
Chemical structure of bioactive phytoconstituent of medicinal plant

Numerous in vivo, and in vitro studies have highlighted the potential of various commonly consumed medicinal plant in combating AD. These plants are rich in bioactive compounds that exhibit significant pharmacological activities, including AChE inhibition, antioxidant and anti-inflammatory effects, neuroprotection, modulation of Aβ toxicity, and regulation of neuronal pathways (Fig. 2). These phytoconstituents found in various medicinal plant that show benefits against AD are detailed in Table 2, with their chemical structures represented in Fig. 3.

**Table 2:** Pharmacological effects of the active phytoconstituents of selected medicinal plant against AD

Plant name	Active compound	Study type	Dose	Pharmacological activity	References
*Allium* *sativum* L	S-allyl-L-cysteine	In vitro*,* (primary mouse hippocampal neurons)	10–1000 ng/mL	Antioxidant, neurotrophic, and anti-ageing cognitive effects	[82]
		In vivo*,* senescence-accelerated SAMP10 mice	20 mg/kg		
	Allicin	In vivo, Aluminum and copper-induced cognitive dysfunction in Wistar rats	10 mg/kg and 20 mg/kg	Improved cognition, decrease oxidative and inflammatory markers, normalized neurotransmitters, and decrease Aβ (1–42)	[83]
Bacopa monnieri (L.) Wettst	Bacopaside I	In vivo, APP/PS1 transgenic mice	15 and 50 mg/kg	Decrease Aβ plaque load and increase learning, memory	[78]
	Bacoside A	In vitro, AChE inhibition assay	1–100 µM	Showed AChE binding/inhibition and neuroprotective potential	[79]
*Boswellia* *sacra*	Incensole acetate (IA)	In vivo*,* Lipopolysaccharide-induced memory impairment in Wistar rats	2.5 and 5 mg/kg	Increase memory while decrease inflammation, oxidative stress and increase BDNF and antioxidants	[84]
	Beta-boswellic acid	In vitro*,* neuronal B65 cell line	1 and 10 μM	Altered CREB-1/CREB-2 expression in a dose- and time-dependent manner, indicating modulation of memory-linked pathways	[85]
*Centella* *asiatica* (L.) Urb	Asiaticoside	In vivo*,* Memory Impairments in Aβ_1-42_-Induced C57/BL6 Mice	40 mg/kg	Improved cognition by blocking p38 MAPK and increase synaptic repair	[86]
*Crocus* *sativus* L	Crocin	In vivo*,* (Aβ_(25–35)_ hippocampal mouse model)	40 mg/kg	Decrease neuroinflammation through PI3K/AKT activation, thereby increase cognitive function	[75]
	Crocetin	In vitro	3.12–50 µM	Increase Aβ clearance, lowers Aβ burden and gliosis, and increase memory, consistent with pro-autophagy neuroprotection	[76]
		in vivo*,* 5XFAD transgenic AD mice	5, 10, 20 mg/kg		
	Safranal	In vivo*,* amyloid-β (Aβ) microinjected rat model	0.025, 0.1, and 0.2 mL/kg	Increase cognition, decrease oxidative stress markers and prevented neuronal loss	[76]
	*trans*-Crocetin	In vitro		Binds Aβ monomers and redirects their aggregation pathway, markedly decrease oligomer and fibril formation	[87]
*Curcuma* *longa* L	Curcumin	In vivo*,* lipopolysaccharide induced C57BL/6 mice	50 mg/kg,	Prevented long-term memory deficits, decrease neuroinflammation and oxidative stress, and preserved recognition memory, and neuroprotective	[88]
*Ginkgo* *biloba* L	Bilobalide	In vivo*,* APP/PS1 AD mice	0.5 mg/kg	Decrease STAT3-linked inflammatory cytokines and increased Aβ-degrading enzymes, decrease amyloid and restoring neuronal function	[89]
*Hericium* *erinaceus*	Hericene A	In vivo	5 mg/kg	Increase memory and activated BDNF, TrkB pathways	[90]
*Melissa* *officinalis* L	Rosmarinic acid	In vivo		Decrease Aβ and p-tau levels and improved memory, likely through JNK suppression and decrease neuroinflammation	[91]
	Luteolin	In vivo	20 and 40 mg/kg	Increase spatial learning and memory while decrease Aβ plaques, astrocyte overactivation, inflammatory cytokines, and ER-stress markers, supporting neuroprotective and anti-inflammatory actions	[92]
		In vitro*,* C6 glial cells	1 and 10 µM		
	Ursolic acid	In vivo	10, 20, 40 mg/kg	Restored Aβ25-35-impaired memory and reduced oxidative and inflammatory markers,	[93]
*Nigella* *sativa* L	Thymoquinone	In vivo	10 and 20 mg/kg	Increase memory performance; decrease hippocampal neuronal loss (CA1); decrease Aβ deposition, phosphorylated-tau and BACE-1 expression; modulated miR, Bax profiles	[94]
*Salvia* *miltiorrhiza* Bunge	Salvianolic acid B	In vitro*,* SH-SY5Y-APPsw Cells	25, 50, 100 µM	Decrease oxidative stress and blocks GSK3β, which may decrease BACE1 expression and amyloid genesis	[95]
	Tanshinone IIA	In vivo*,* APP/PS1 transgenic mice	25–100 mg/kg	Improves cognitive decline and neuroinflammation by partly inhibiting the RAGE, NF-κB pathway	[96]
*Vitis* *vinifera* L	Vitisin A	A human neuroblastoma cell line, SH-SY5Y cells	1, 5, or 10 μM	Increase cognitive task performance, increase cell survival under oxidative stress, and increase hippocampal BDNF–CREB signaling	[97]
		scopolamine-induced in dementia C57BL/6 male mice model	1 or 100 ng/μL

In addition to pharmacodynamic activity, the pharmacokinetic characteristics of key bioactive constituents critically influence their translational potential. Curcumin exhibits extremely low oral bioavailability due to poor aqueous solubility, rapid intestinal metabolism, and extensive first-pass hepatic clearance; however, nanoparticle, liposomal, phospholipid-complex, and bioenhancer-based formulations significantly improve systemic exposure and brain distribution [69–74]. Crocin displays limited gastrointestinal absorption and is rapidly hydrolyzed to crocetin, which shows superior membrane permeability, measurable blood–brain barrier penetration, and a longer elimination half-life, supporting its role as the principal bioavailable neuroactive metabolite of saffron [46, 47, 75–77]. Emerging pharmacokinetic studies of asiaticoside, bacosides, bilobalide, huperzine A, and erinacines further indicate variable absorption, metabolism, and brain exposure, emphasizing the importance of formulation strategies for optimized central nervous system delivery [71, 75, 78–81].

### *Allium sativum* L.

*A.* *sativum* L. (garlic), a member of the Amaryllidaceae family, has long been used as both a culinary ingredient and a medicinal agent, with traditional applications ranging from respiratory and gastrointestinal ailments to hypertension, hyperlipidemia and general cognitive vitality [98]. Its historical use across Asia, Europe and the Middle East for inflammatory and circulatory disorders is particularly notable, as these conditions are now recognized contributors to neurodegenerative disease risk [98]. The pharmacological actions of garlic are primarily attributed to its organosulfur constituents including allicin and S-allyl-L-cysteine alongside flavonoids and other antioxidant molecules, which together exert antioxidative, anti-inflammatory, lipid-regulating and anti-amyloidogenic effects that support a mechanistic rationale for neuroprotection [98]. Evidence from preclinical studies shows that garlic extracts and isolated compounds reduce oxidative stress, inhibit acetylcholinesterase (AChE), modulate apoptosis-related markers (Bax/Bcl-2, caspase-3), suppress neuroinflammation and decrease amyloid-β burden, accompanied by improvements in learning and memory in rodent models [99, 100]. Aged garlic extract (AGE) and stabilized allicin formulations, developed to improve bioavailability and tolerability, have demonstrated the ability to preserve mitochondrial function and maintain synaptic proteins under Aβ-induced stress in both in vitro and in vivo assays [99, 100]. Consistent with these findings, essential oil derived from garlic cloves showed moderate anti-cholinesterase activity in vitro, inhibiting AChE (65.4%) and BChE (31.5%), with dose-dependent effects on cholinergic markers [33]. In Aβ_(25–35)_-induced AD rodent models, garlic administration increased hippocampal brain-derived neurotrophic factor (BDNF) and reduced AChE activity, supporting its neurotrophic potential [34]. S-allyl-L-cysteine further enhanced neurite outgrowth in primary mouse hippocampal neurons at 10–1000 ng/mL and improved cognitive performance in senescence-accelerated SAMP10 mice while preserving AMPA/NMDA receptor expression and p-CaMKII signaling [82]. Allicin supplementation (10–20 mg/kg) attenuated aluminum- and copper-induced cognitive deficits in Wistar rats, lowered oxidative and inflammatory markers (IL-1β, IL-6, TNF-α), restored neurotransmitter balance, and reduced Aβ(1–42) accumulation in a dose-dependent manner [83].

### *Bacopa monnier* (L.) Wettst.

*B.* *monnieri* (brahmi, water hyssop) is a perennial creeping herb of the *Plantaginaceae* family, widely valued in Ayurvedic medicine as a cognitive enhancer and nervine. Traditionally, it has been used to improve memory, reduce anxiety, support sleep, and manage epilepsy, with additional ethnobotanical reports noting benefits for asthma, digestive discomfort, and wound healing [101, 102]. Contemporary ethnopharmacological research supports these long-standing uses and highlights growing interest in bacosides and related constituents for their antioxidant, anti-inflammatory, cholinergic, and synaptic-supportive effects, all of which are relevant to neurodegenerative disorders [101, 102]. Pharmaceutical development has focused on standardized bacoside-rich extracts used as nootropic supplements, isolation of bacoside A and other saponins as potential lead molecules, and formulation strategies including nanoparticle and liposomal carriers designed to enhance oral absorption and overcome poor solubility and first-pass metabolism. Combination products with other neuroprotective agents have also been explored to assess possible synergistic activity [38, 103]. Alzheimer’s-focused preclinical studies consistently show that *B.* *monnieri* exerts broad neuroprotective actions. In rodent and cell-based AD models, extracts reduce amyloid-β–induced toxicity, limit oxidative stress and neuroinflammation, attenuate tau-related pathology, and improve learning and memory performance in behavioural assays such as the Morris water maze [38, 102]. Mechanistic investigations report enhanced expression of synaptic markers, modulation of acetylcholinesterase activity, and reduced apoptotic signalling, indicating complementary actions that align well with key features of Alzheimer’s pathology [38, 101]. Individual studies further demonstrate that ethanolic extracts of the aerial parts protect against Aβ_42_-induced hippocampal damage at concentrations ranging from 0.25 to 400 μM, improving cognition, lowering oxidative and inflammatory markers, reducing cholinesterase activity, decreasing tau and amyloid pathology, and regulating the GSK-3β/Wnt/β-catenin pathway [36]. In colchicine-induced dementia models, a 50 mg/kg dose improved cognitive outcomes, decreased ROS and NO levels, reduced IL-6, TNF-α, and MCP-1, and lowered BACE-1 activity and amyloid production [37]. An aqueous extract also showed protective effects in IMR-32 neuroblastoma cells exposed to Aβ_42_ at 100 μg/mL [38]. Among isolated constituents, bacopaside I (15–50 mg/kg) reduced amyloid plaque burden and improved memory in APP/PS1 transgenic mice [78], while bacoside A (1–100 μM) demonstrated acetylcholinesterase inhibitory activity and neuroprotective potential in vitro [79].

### *Centella asiatica* (L.) Urb.

*C.* *asiatica*, widely known as gotu kola and pennywort and belonging to the Apiaceae family, is a perennial herb valued for centuries in Ayurvedic, Traditional Chinese and Southeast Asian systems of medicine for its wound-healing, anxiolytic and cognition-enhancing effects [81]. Traditional uses include whole leaves and aqueous preparations for the management of skin wounds and ulcers, gastrointestinal discomfort, anxiety, age-related memory complaints and general vitality, and ethnobotanical studies continue to report its recognition as a “memory herb” across regions of India, China and Indonesia [104, 105]. Its therapeutic profile is linked mainly to triterpenoid saponins such as asiaticoside, madecassoside, asiatic acid and madecassic acid, together with polyphenolic constituents, which act through antioxidant, anti-inflammatory, neuroprotective and tissue-repair mechanisms that support both external and internal applications [106]. Modern pharmaceutical development has expanded these traditional uses into standardized aqueous extracts designed for cognitive support, topical preparations for wound healing and scar reduction, nutraceutical formulations enriched with triterpenoid fractions and emerging pharmacokinetic studies aimed at defining exposure, safety and dosage for human use [107, 108]. Extensive preclinical evidence has examined the potential of *C.* *asiatica* in Alzheimer’s disease models. Aqueous extracts and defined constituents have been shown to regulate oxidative stress pathways, enhance mitochondrial biogenesis, activate Nrf2-dependent antioxidant gene networks and shift metabolic pathways associated with amyloid burden in transgenic models such as 5×FAD mice, leading to improved learning and memory across behavioural tasks [106]. Studies isolating fractions enriched in caffeoylquinic acids or specific triterpenoid components report reversal of cognitive deficits and reduction of neuropathological features in mouse systems, highlighting both symptomatic and potential disease-modifying actions [109, 110]. These findings are supported by validated analytical methods and an early-phase human pharmacokinetic and safety study conducted in older adults, marking important steps toward clinical translation and reinforcing the need for extract standardization and dose selection guided by animal data [107]. Additional experimental work using water extracts of *C.* *asiatica* leaves and stems at 200 and 1000 mg/kg in aged transgenic AD mice (Tg2576) and wild-type controls has demonstrated improvements in novel object recognition and contextual fear memory, changes in hippocampal metabolomic profiles, enhanced mitochondrial respiration and reductions in oxidative stress markers [40]. Ethanol extracts tested in neuronal cell lines such as PC12 and IMR32 at concentrations of 25, 50 and 100 μg/mL protected against Aβ1-40–induced toxicity, lowered reactive oxygen species and strengthened endogenous antioxidant defences including superoxide dismutase, catalase and glutathione systems [41]. In 5×FAD mice, daily treatment with water extracts at 200, 500 and 1000 mg/kg improved object recognition and contextual fear performance, increased expression of Nrf2-regulated antioxidant genes and reduced oxidative stress indicators [42]. Asiaticoside at 40 mg/kg in Aβ1-42-induced C57/BL6 mice alleviated memory impairment by inhibiting p38 MAPK activation and promoting synaptic repair, with evidence of reduced neuroinflammation and improved synaptic function, suggesting strong potential for therapeutic development [86]

### *Crocus sativus* L.

*C.* *sativus* widely known as saffron and belonging to the family Iridaceae, is a perennial autumn-flowering bulb whose dried stigmas have been valued for millennia as a spice, dye, and medicinal substance throughout Mediterranean, Persian, Indian, and East Asian medical traditions. Across these long-standing records, saffron has been used for diverse conditions including mood disturbances, insomnia, menstrual irregularities, digestive problems and respiratory complaints, and historical writings together with ethnobotanical research also describe its application for memory and age-related cognitive decline [111]. Current pharmacological research attributes its central nervous system activity to major constituents such as crocin, crocetin, picrocrocin and safranal, which exhibit antioxidant, anti-inflammatory, anti-apoptotic and neurotransmitter-modulating actions that are mechanistically consistent with neuroprotection [112, 113]. Pharmaceutical development efforts increasingly focus on these compounds: crocin and crocetin are being evaluated as neuroprotective nutraceuticals and standardized botanical preparations for cognitive disorders; safranal is under investigation as a small-molecule modulator of GABAergic and monoaminergic signalling with potential antidepressant and anxiolytic benefits; standardized saffron extracts are being formulated for oral delivery to target mild cognitive impairment and reduce dementia risk in clinical settings; and derivative formulations such as by-products and encapsulated crocin complexes are being explored to improve stability, bioavailability and blood brain-barrier permeability for neurological indications [77, 113]. Converging preclinical evidence highlights robust multi-target effects relevant to AD. Studies in rodents and cell systems show that saffron extracts and isolated crocin attenuate amyloid-β toxicity, limit oxidative and inflammatory responses, inhibit cholinesterase activity, regulate autophagy and apoptosis pathways, and strengthen synaptic plasticity, leading to improvements in learning and memory across behavioral tasks such as the Morris water maze and passive avoidance tests [47, 77]. Importantly, these mechanisms do not act independently; rather, antioxidant activity appears to synergize with anti-inflammatory signaling by reducing reactive oxygen species–driven activation of inflammatory pathways such as NF-κB and NLRP3, thereby amplifying neuroprotection. This coordinated suppression of oxidative stress and neuroinflammation contributes to downstream stabilization of mitochondrial function, preservation of synaptic integrity, and reduced amyloidogenic processing, illustrating a tightly interconnected multi-target therapeutic network [47, 77]. In transgenic 5×FAD mice, a hydroalcoholic extract of *C.* *sativus* given at 50 mg/kg per day improved blood–brain-barrier integrity, increased amyloid-β clearance through P-gp and LRP1, reduced amyloid burden, up-regulated synaptic proteins and lowered neuroinflammation [46]. The enhancement of amyloid clearance is mechanistically linked to concurrent reductions in oxidative and inflammatory stress, which are known to impair transporter function at the blood–brain barrier, suggesting that antioxidant and anti-inflammatory actions indirectly facilitate amyloid efflux [46]. Complementary in vitro work using the same extract demonstrates enhanced amyloid-β clearance through P-gp induction, inhibition of the NLRP3 inflammasome with reduced IL-1β release, and protection of SH-SY5Y cells against amyloid-β and glutamate toxicity, alongside behavioral improvement in streptozotocin and scopolamine models [47]. Additional in vivo studies in streptozotocin-induced dementia in rats and scopolamine-induced amnesia in mice further support these effects at doses of 50 mg/kg and 100 mg/kg [47], while chronic scopolamine-treated rats show significant recovery of memory at 10, 15 and 20 mg/kg with reduced acetylcholinesterase activity, lower IL-6 and oxidative stress, and a reduction in amyloid plaques and neurofibrillary tangles [49]. Here, cholinesterase inhibition may act synergistically with anti-inflammatory and antioxidant effects to sustain cholinergic neurotransmission while minimizing cytokine- and oxidative-mediated synaptic damage [49] Crocin administered at 40 mg/kg in a hippocampal amyloid-β25–35 mouse model suppresses neuroinflammation through activation of the PI3K, AKT pathway, improving cognitive outcomes [75]. Crocetin at concentrations of 3.12–50 µM enhances amyloid clearance in vitro, lowers brain amyloid load and gliosis, and improves memory in 5XFAD mice, indicating autophagy-promoting and neuroprotective effects at doses of 5, 10 and 20 mg/kg in vivo [76]. Safranal given at 0.025, 0.1 and 0.2 mL/kg in an amyloid-β microinjected rat model improves cognitive performance across multiple tasks including the Y-maze, novel-object recognition, passive avoidance and radial arm maze, while lowering oxidative, inflammatory and apoptotic markers and preventing neuronal loss, demonstrating antioxidant, anti-inflammatory and anti-apoptotic protection [114]. The convergence of these pathways suggests that suppression of apoptosis is secondary to upstream control of oxidative stress and inflammation, reinforcing the concept of hierarchical and synergistic mechanism integration [114]. Finally, trans-crocin 4 and trans-crocetin have been shown in vitro to bind strongly to amyloid-β monomers and redirect aggregation pathways, markedly reducing oligomer and fibril formation, which points to a direct anti-amyloid mechanism for these specific components [87].

### *Curcuma longa* L.

*C.* *longa* widely known as turmeric, is a perennial herb in the ginger family Zingiberaceae and has long been valued in South Asian traditional medicine for its broad therapeutic uses. The rhizomes have historically been applied as a wound poultice and used as an anti-inflammatory agent, digestive tonic, and treatment for liver, respiratory, and skin disorders; in Ayurvedic and folk traditions they have also been taken as a memory tonic and for age-related cognitive decline [69]. Modern phytochemical and pharmacological studies have centred on curcumin, the principal diferuloylmethane polyphenol, whose wide biological actions including antioxidant, anti-inflammatory, metal-chelating, and cell-signalling modulatory effects provide a plausible basis for many longstanding medicinal claims [70, 71]. Pharmaceutical development has advanced rapidly, with curcumin and turmeric extracts now explored as nutraceuticals and supportive treatments for chronic inflammation, metabolic dysfunction, and neurodegenerative diseases. Recent formulation research has focused on improving oral bioavailability through nanoparticle, liposomal, phospholipid, and polymer-based delivery systems; combining curcumin with bioenhancers such as piperine to increase systemic exposure; designing curcumin analogues and prodrugs with greater metabolic stability; and producing standardized turmeric extracts to ensure consistent dosing in clinical studies all intended to convert promising laboratory findings into reliable clinical benefit [69, 70]. Preclinical research provides strong and consistent evidence that curcumin can reduce several core pathological processes in animal and cell models. It decreases amyloid-β aggregation and deposition, suppresses neuroinflammation by modulating microglial and astrocyte activity, lowers oxidative stress, inhibits pathways linked to tau hyperphosphorylation, and supports synaptic plasticity and neurotrophic signalling, including effects on BDNF. These actions have been associated with improved spatial memory and learning in both transgenic and toxin-induced rodent models [72–74]. Experimental studies using turmeric extracts further reinforce these findings. Ethanol extracts of the rhizome administered to amyloid-β_1–42_-injected mice at 100 and 300 mg/kg reduced Aβ-induced cytotoxicity, lowered TNF-α, IFN-β, and iNOS, inhibited TLR4–NF-κB signalling, and improved memory and learning in Morris water maze and passive avoidance tests; complementary in vitro work in primary rat hippocampal neurons at 3–30 µg/mL supported these anti-inflammatory and neuroprotective effects [43]. Methanolic extracts of the rhizome showed strong inhibition of amyloid-fibril formation in a cell-free fibrillation model, indicating potential to limit amyloid aggregation [44]. Additional ethanol-based extracts demonstrated protective, antioxidant, and anti-inflammatory actions in C6 glioma and BV2 microglia, with parallel improvements in scopolamine-induced amnesia in mice at doses of 50, 100, and 200 mg/kg, highlighting relevance to neurodegeneration [45]. Curcumin itself prevented long-term memory deficits in lipopolysaccharide-induced C57BL/6 mice at 50 mg/kg, reduced neuroinflammation and oxidative stress, and preserved recognition memory, suggesting meaningful protection against inflammatory drivers of Alzheimer’s pathology [88].

### *Ginkgo biloba *L.

*G.* *biloba* widely known as ginkgo or the maidenhair tree, is the only surviving species of the ancient gymnosperm family Ginkgoaceae and has been valued for centuries in East Asian medicine for disorders of memory, poor circulation, respiratory symptoms, and urinary complaints. Its leaves and seeds were traditionally used to relieve asthma, bronchitis, intermittent claudication, and were often regarded as a general tonic for the brain [115]. Contemporary ethnopharmacological reports continue to highlight the leaf extract for cognitive difficulties and age-related memory decline, which stimulated systematic investigation of ginkgo for Alzheimer’s disease (AD) and other dementias [116, 117]. The most rigorously studied preparation is the standardized extract EGb 761^®^, formulated to contain about 24% flavonol glycosides and about 6% terpene trilactones, including ginkgolides and bilobalide, while maintaining a low concentration of ginkgolic acids. This standardized composition forms the basis of most clinical and preclinical research and explains the extract’s consistent antioxidant, anti-inflammatory, and vascular actions [118]. In pharmaceutical use, EGb 761^®^ is produced as oral tablets or capsules for cognitive support and for the symptomatic management of mild to moderate dementia; it is used clinically to enhance cerebral blood flow and microcirculation; its flavonoids act as free-radical scavengers, while its terpene lactones influence platelet-activating factor and mitochondrial function; and study protocols commonly use doses between 120 and 240 mg per day, depending on the therapeutic indication. Preclinical AD studies have consistently shown that ginkgo leaf extracts and EGb 761^®^ exert neuroprotective activity across cellular and transgenic mouse models. These preparations reduce amyloid-β aggregation and tau-related toxicity, limit oxidative stress and neuroinflammation, preserve mitochondrial integrity and synaptic markers, and improve performance in behavioral tests of learning and memory. Mechanistic analyses link these effects to the induction of antioxidant enzymes, suppression of excitotoxic glutamate release, regulation of autophagy and lysosomal pathways, and a reduction in microglial pro-inflammatory signaling [119]. Experimental data support these findings: in TgCRND8 mice, 240 mg/day of EGb 761 improved cognition in the Barnes maze, prevented the loss of synaptic proteins such as PSD-95, Munc18-1, and SNAP25, and reduced neuroinflammation [50]. In APP, PS1 mice, 100 mg/kg of a standardized extract improved spatial memory in the Morris water maze and altered gut microbial composition by increasing probiotic species and modifying metabolite profiles [51]. In SH-SY5Y human neuroblastoma cells, 25 µg/mL of leaf extract reduced calcium influx through TRPV1 channels, limited reactive oxygen species, apoptosis, and mitochondrial depolarization in an AD model [48]. Bilobalide, a major terpene trilactone of the plant, showed complementary benefits in APP, PS1 mice at 0.5 mg/kg by lowering STAT3-dependent TNF-α, IL-1β, and IL-6 signaling, increasing amyloid-β-degrading enzymes such as NEP, IDE, and MMP2, diminishing amyloid burden and inflammation in brain tissue, and ultimately restoring neuronal integrity [89].

### Hericium erinaceus

*H.* *erinaceus*, widely known as Lion’s mane and belonging to the family Hericiaceae, is an edible toothed fungus long used in East Asian medicine for digestive problems, gastric ulcers, general restorative purposes, and, most notably, for traditional claims that it can strengthen the nerves and improve memory [120, 121]. Recent scientific assessments increasingly support these long-standing uses, linking them to specific groups of bioactive molecules hericenones derived from the fruiting body and erinacines produced by the mycelium which interact with neurotrophic and anti-inflammatory pathways and have therefore become the focus of preclinical research in Alzheimer’s disease (AD) [120, 122]. Pharmaceutical interest in *H.* *erinaceus* continues to grow due to its neurotrophic activity and favorable safety profile. Current developments include standardized erinacine-A–rich mycelial extracts designed for oral nutraceuticals that promote nerve growth factor (NGF); isolated hericenone fractions studied as small-molecule candidates to stimulate neurite outgrowth; polysaccharide preparations explored for anti-inflammatory actions and microbiome-mediated brain effects; combined extracts from both mycelium and fruiting bodies formulated for long-term dietary supplementation; and formulation strategies aimed at improving the oral absorption and central nervous system penetration of erinacines [120, 123].

Preclinical AD studies have produced consistent evidence that extracts and purified compounds from *H.* *erinaceus* can reduce several core mechanisms implicated in Alzheimer’s pathology. In cellular and rodent models, erinacine A increases NGF expression, promotes neurite extension, reduces amyloid-β formation and amyloidogenic processing, and decreases markers of inflammation such as IL-1β, while also reducing oxidative stress and protecting neural cells from amyloid-β toxicity. These changes lead to better performance on learning and memory tasks including the Morris water maze and novel object recognition in multiple mouse studies [120, 121, 124]. Standardized mycelial extracts have also been shown to lower amyloid plaque load and preserve synaptic markers in APP, PS1 and SAMP8 models, supporting a broad disease-modifying role rather than a narrow symptomatic effect [124, 125]. Although small clinical studies and short-term supplementation trials in older adults and individuals with mild cognitive impairment have produced mixed yet encouraging cognitive outcomes, larger and well-controlled randomized trials using defined extracts, dose–response evaluation, and extended follow-up are still necessary to determine the therapeutic potential, optimal dosing, and preventive value of *H.* *erinaceus* for AD [126, 127]. Experimental work further supports these findings. Mycelial aqueous extracts protected PC12 cells from glutamate-induced apoptosis at 50 and 100 µg/mL by lowering reactive oxygen species, preventing calcium overload, and reducing mitochondrial dysfunction. In mice, the same extract improved locomotor activity, enhanced rotarod endurance, reduced escape latency in the Morris water maze, and increased acetylcholine and choline acetyltransferase levels in serum and the hypothalamus in an aluminum chloride and d-galactose model of AD [52]. Ethanolic extracts tested in scopolamine-induced zebrafish models at 0.5, 1, and 3 mg/L improved behavioral memory outcomes, inhibited acetylcholinesterase activity, strengthened antioxidant defenses (SOD, CAT, GPX, GSH), and reduced lipid peroxidation and protein–carbonyl damage in the brain [53]. Aqueous preparations from whole biomass administered at 200 mg/kg up-regulated vitagene stress-response proteins, including Hsp70, HO-1, and thioredoxin, while increasing lipoxin A4 across the cortex, cerebellum, and hippocampus, indicating reduced neuroinflammation and activation of endogenous resolution pathways relevant to AD [54]. In addition, purified hericene A given at 5 mg/kg improved recognition memory and activated broad neurotrophic signaling, including BDNF and TrkB, strengthening synaptic function and positioning the hericene/erinacine family as promising neuroprotective candidates for AD-related cognitive decline [90].

### *Huperzia serrata* (Thunb.) Trevis.

*H.* *serrata*, commonly known as Chinese club moss or toothed clubmoss, is a slow-growing lycopod belonging to the family Lycopodiaceae and has a long history of use in traditional Asian medicine for conditions such as memory loss, fever, inflammation, cough, and trauma. Ethnobotanical accounts describe its valued role in Traditional Chinese Medicine and related practices, where whole-plant preparations were regarded as agents that support healthy ageing and cognitive function [128]. Modern phytochemical research has identified the plant’s principal bioactive alkaloid, huperzine A (HupA), as a potent, reversible, and selective acetylcholinesterase inhibitor, providing a mechanistic explanation for its traditional use in forgetfulness and age-related cognitive decline [80, 129]. In pharmaceutical development, HupA has been advanced both as a symptomatic cholinergic therapy enhancing acetylcholine levels and improving short-term cognition and as a molecule with broader neuroprotective properties that include antioxidant actions, anti-apoptotic signaling, modulation of amyloid precursor protein processing, and protection of synaptic integrity [81, 129]. Current applications focus on oral HupA formulations for dementia symptom relief, supported by animal models and reported clinical benefits. Preclinical studies also indicate antioxidant, anti-apoptotic, and mitochondrial-stabilizing effects that may slow neuronal loss, while emerging data on amyloid and tau modulation suggest multi-target, disease-modifying potential beyond acetylcholinesterase inhibition. Between 2020 and 2024, structural-modification and drug-delivery studies have sought to improve brain bioavailability and safety, and recent regulatory reviews emphasize the importance of dose-finding and standardization for herbal preparations [80, 81]. Standardized extracts of *H.* *serrata* and purified HupA consistently reverse or attenuate learning and memory impairments in rodent models, reduce acetylcholinesterase activity in the hippocampus and cortex, lower oxidative stress markers, and influence biomarkers linked to amyloid metabolism and synaptic function. These findings, summarized across recent preclinical reviews and individual experimental studies, support continued translational interest while also highlighting the need for rigorous dose–response designs, long-term safety evaluation, and standardized extract parameters before broad clinical implementation [80, 81, 129]. Experimental data show that aqueous or ethanolic extracts at 30 mg/kg/day inhibit acetylcholinesterase in the brain and improve cognitive performance in scopolamine-induced mice using Y-maze and passive avoidance tests [55]. In SH-SY5Y cells exposed to scopolamine, extracts promote neuroprotection through increased antioxidant enzyme activity (SOD, CAT), decreased malondialdehyde, reduced acetylcholinesterase activity, and improved behavioral outcomes in aligned in vivo assessments [56]. Additional studies in scopolamine-treated mice using Y-maze and the Morris water maze further support the extract’s ability to improve learning and memory, consistent with its proposed neuroprotective and cholinergic effects[56].

### *Salvia miltiorrhiza* Bunge

*S.* *miltiorrhiza* Bunge, widely known as Danshen or red sage, is a perennial herb from the mint family Lamiaceae whose dried roots have long held a central role in East Asian traditional medicine. In traditional Chinese medicine, Danshen is used to invigorate blood and resolve blood stasis, and it continues to be prescribed for cardiovascular and cerebrovascular disorders such as angina, ischemia, and stroke, as well as for menstrual disturbances, hepatic conditions, and inflammatory diseases [130]. Advances in phytochemistry have defined two major groups of active molecules: lipophilic tanshinones, including tanshinone IIA and cryptotanshinone, and water-soluble phenolic acids, such as salvianolic acids. Together, these compounds account for the herb’s recognized circulatory, antioxidant, and anti-inflammatory actions [131]. Danshen is developed into standardized oral and injectable products enriched in tanshinones and salvianolic acids, and preparations such as compound Danshen dripping pills and Danshen injections are used clinically in China for ischemic cardiovascular disease. Its bioactive molecules are being engineered into nano-delivery systems to enhance delivery to the brain and to tumors, tanshinone IIA is under investigation as a lead compound for central nervous system drug development, salvianolic acids are studied as antioxidant and anti-amyloid candidates, and cryptotanshinone shows activity in key cell-signaling pathways with relevance to neuroprotection. Ongoing regulatory and quality-control work emphasizes standardization and characterization of drug–herb interactions to support clinical translation [132, 133]. Preclinical studies increasingly examine Danshen and its constituents in Alzheimer’s disease models. Tanshinone IIA and related diterpenoids have been shown to lower amyloid-β burden, protect against amyloid-β-induced cytotoxicity, suppress neuroinflammation through NF-κB signaling, and reduce oxidative stress and neuronal apoptosis in both cell-based systems and transgenic or chemically induced rodent models, with parallel improvements in learning and memory performance [134, 135]. Salvianolic acids demonstrate strong antioxidant capacity and inhibit amyloid-β aggregation in vitro, while improving synaptic and mitochondrial markers in animal studies; cryptotanshinone also modulates signaling pathways implicated in neurodegeneration and provides neuroprotective effects in preclinical evaluations [136, 137]. Experimental work using the ethanol extract of *S.* *miltiorrhiza* root in NG108-15 cells and in mice exposed to amyloid-β_(25–35)_ shows attenuation of learning and memory deficits in Y-maze and passive-avoidance tasks, along with reduced brain malondialdehyde levels, with effective doses ranging from 400 to 1200 mg/kg in vivo and 2.0 μg/mL in vitro [63]. The dried root and rhizome water extract binds amyloid-β, inhibits fibril formation in Thioflavin T assays, delays amyloid-β-induced paralysis in Caenorhabditis elegans, decreases reactive oxygen species generation by more than 50 percent at 4 mg/mL, and provides antioxidant and neuroprotective activity against amyloid-β-mediated toxicity [64]. In a chronic cerebral hypoperfusion model, root and rhizome water extract at 200 mg/kg per day protects hippocampal and white-matter integrity following bilateral carotid occlusion, reduces pro-inflammatory cytokines including TNF-α, IL-1β, and IL-6, inhibits the TLR4-MyD88 pathway, and restores myelin basic protein levels [65]. Salvianolic acid B reduces oxidative stress and inhibits GSK3β activity in SH-SY5Y-APPsw cells at concentrations of 25–100 μM, an effect linked to suppression of BACE1 expression and reduced amyloidogenesis [95]. Tanshinone IIA, administered at 25–100 mg/kg in APP/PS1 transgenic mice, improves cognitive impairment and decreases neuroinflammation, in part through inhibition of the RAGE–NF-κB signaling pathway [96].

### *Vitis vinifera* L.

*V.* *vinifera*, commonly known as grape and belonging to the Vitaceae family, has been valued in traditional medical systems for centuries, where grape fruit, seeds, skins and leaves were used to support digestion, promote wound healing and enhance memory, often described culturally as a “brain tonic” that conveys vitality and cognitive strength. Contemporary phytochemical research confirms that these plant parts contain abundant polyphenols such as resveratrol, proanthocyanidins, anthocyanins and flavonols, compounds that provide a credible scientific basis for these long-standing ethnomedical claims and explain why *V.* *vinifera* now holds a prominent position in nutraceutical investigations targeting age-related cognitive decline. Preclinical studies in AD models show that preparations derived from this species exert diverse neuroprotective effects, including strong antioxidant and free-radical-scavenging activity, suppression of neuroinflammation, inhibition of amyloid beta aggregation and tau pathology, enhancement of synaptic plasticity through BDNF–CREB signaling, and support of mitochondrial function mechanisms demonstrated across multiple cellular systems and rodent AD models and synthesized in recent reviews [138, 139]. In pharmaceutical and translational contexts, compounds from *V.* *vinifera* are being advanced as multimodal agents or as adjuncts to enhance existing therapies rather than as single-target interventions. Current applications include standardized grape-seed polyphenol extracts formulated as oral nutraceuticals for cognitive maintenance; resveratrol analogs designed as SIRT1-activating small molecules with improved bioavailability; nanoparticle and liposomal carriers engineered to increase brain penetration of grape polyphenols; topical preparations from leaves and skins used in antioxidant skincare and wound-healing approaches that draw upon shared anti-inflammatory chemistry; and combination formulations that pair grape polyphenols with other neuroprotective phytochemicals to achieve synergistic effects relevant to AD modulation [140]. In Alzheimer’s research specifically, whole grape extracts, grape-seed extract (GSPE), anthocyanin-rich fractions and purified resveratrol have been shown to improve cognitive performance in behavioral paradigms, reduce amyloid burden and tau-related pathology, restore antioxidant enzyme activities and rescue synaptic markers in various experimental models, including scopolamine-, D-galactose- and amyloid beta–based systems as well as transgenic animals, alongside protection demonstrated in cellular assays that assess amyloid-induced toxicity [141]. Early human supplementation studies using standardized grape extracts or resveratrol also report promising cognitive and biomarker responses that support further translational work, although experts consistently highlight the need for larger, well-controlled clinical trials and improved formulations to address the low oral bioavailability and rapid metabolism that limit many grape polyphenols [138, 142]. Experimental data further reinforce these findings: petroleum ether leaf extracts at 100 mg/kg in an aluminum-induced rat model of Alzheimer’s improved cognitive and neurobehavioral outcomes and modulated markers including acetylcholine, acetylcholinesterase, BDNF, IL-6 and homocysteine, demonstrating neuroprotective, antioxidant, anti-inflammatory and anti-amnesic activity [66]. Seed extracts administered at 250 and 500 mg/kg in the same model improved learning and memory in the Morris water maze, inhibited mRNA expression of APP and tau, reduced lipid peroxidation and inflammation, and strengthened cholinergic function [67]. Ethanol extracts from the leaves protected HT22 hippocampal neuronal cells against glutamate-induced oxidative stress at 50 μg/m by enhancing antioxidant enzyme gene expression, including SOD, CAT and GPx [68]. Vitisin A, a constituent of Vitis vinifera, improved cognitive performance in behavioral assessments, increased cell survival under oxidative stress in SH-SY5Y neuroblastoma cells and enhanced hippocampal BDNF–CREB signaling at concentrations of 1, 5 or 10 μM, with supportive findings also reported in scopolamine-induced dementia models in C57BL/6 male mice at doses of 1 or 100 ng/μL [97].

### Clinical trials of medicinal plant against AD

Building on the promising outcomes from various studies, many medicinal plants have progressed to clinical trials to assess their efficacy and safety in the prevention and treatment of AD in humans. The diverse pharmacological effects observed in these trials underscore the potential of medicinal plants in AD prevention and treatment (Table 3). A growing body of clinical evidence indicates that several medicinal plants may support cognitive function across a range of populations, from healthy adults to individuals with mild cognitive impairment (MCI), Alzheimer’s disease (AD), and vascular dementia. In healthy volunteers, supplementation with *A.* *sativum* for five weeks improved visual memory and attention, although changes in verbal memory and executive function did not reach statistical significance, suggesting that longer trials in neurodegenerative conditions may better reveal its protective effects [143]. In patients with MCI and mild-to-moderate AD, *B.* *sacra* administered for three months produced meaningful improvements in cognitive and behavioral symptoms, reinforcing its therapeutic potential in early cognitive decline [144]. A phase-1 crossover study of *C.* *asiatica* showed that single oral doses of a standardized aqueous extract were safe and well tolerated, with active compounds detected in plasma and evidence of dose-dependent antioxidant and neuroprotective activity through NRF2 pathway activation in individuals with MCI [107]. However, this study enrolled only four participants with mild cognitive impairment, which substantially limits statistical power and precludes meaningful assessment of interindividual variability or clinical efficacy. While valuable for confirming safety, pharmacokinetics, and mechanistic signals such as NRF2 pathway activation, the extremely small sample size restricts extrapolation of these findings to broader AD populations and underscores the need for larger, adequately powered follow-up trials [107]. *C.* *sativus* demonstrated clinical efficacy comparable to donepezil during a 22-week randomized trial in mild-to-moderate AD, highlighting its potential as a botanical alternative for symptomatic treatment [145]. *G.* *biloba* extract (EGb 761) improved executive function, attention, non-verbal memory, mood, and quality of life in brain tumor survivors with radiation-induced cognitive dysfunction, although a high dropout rate suggested tolerability and perceived benefit may vary across individuals [146]. Long-term supplementation with *H.* *erinaceus* mycelia over 49 weeks slowed cognitive decline in mild AD and improved daily function and contrast sensitivity, with a strong safety profile supporting its use as a neuroprotective agent [147]. *H.* *serrata* also showed clinical value, reducing cognitive and task-switching deficits over an eight-week period in patients with AD [148]. In a 96-week double-blind trial, *M.* *officinalis* prevented cognitive decline in individuals with mild AD dementia, further affirming its long history of cognitive use [149]. Among healthy elderly adults, *N.* *sativa* taken for nine weeks enhanced memory, attention, and general cognition, suggesting possible preventive effects against age-related cognitive decline or early neurodegenerative processes [150]. *P.* *ginseng* improved MMSE and ADAS scores in patients with AD over a 12-week treatment period, though the gains diminished after discontinuation, indicating that continuous intake may be necessary to sustain cognitive benefits [151]. For vascular dementia, *S.* *miltiorrhiza* showed both efficacy and safety over a 24-week multicenter trial, supporting its potential role in managing cognitive impairment linked to vascular dysfunction [152]. In healthy older adults, *V.* *vinifera* extract (Cognigrape^®^) administered for 12 weeks improved memory, attention, and language skills, suggesting a favorable effect on age-related cognitive performance [153]. A multi-herb formulation, Yi-Zhi-An-Shen granules, prevented or delayed cognitive decline over 16 weeks in individuals with MCI or dementia, reflecting the therapeutic value of synergistic plant combinations [154]. Similarly, a polyherbal formulation containing *B.* *monnieri*, *H.* *rhamnoides*, and *D.* *bulbifera* improved cognition and reduced inflammation and oxidative stress in senile dementia of the Alzheimer’s type following one year of treatment [155]. A short-term crossover study combining *P.* *ginseng*, *G.* *biloba*, and *C.* *sativus* reported enhanced visuospatial short-term memory, working memory, and more efficient attentional processing, aligning with earlier preclinical results and underscoring strong synergistic interactions among these botanicals [156]. Finally, the Korean herbal medicine Kami Guibi-tang showed positive cognitive effects in patients with mild AD over a 24-week trial, indicating its promise for individuals in the early stages of impairment [157].

**Table 3:** Clinical trials investigating the use of medicinal plant for preventing and treating AD

Plant name	Study type	Administered material	Duration	Number of patients	Health condition of patients	Dose	Result	References
*Allium* *sativum* L	Randomized, placebo-controlled Study	Capsule	5 weeks	20	Healthy human volunteers	400 mg twice daily	Increase visual memory and attention	[143]
*Boswellia* *sacra* Flueck	Double-blinded, placebo-controlled trial	Capsule	3 months	60	Patients with MCI and mild-to-moderate AD	500 mg Three times a day	Improved MMSE and reduced neuropsychiatric inventory (NPI) scores, improving cognitive and behavioral symptoms	[144]
*Centella* *asiatica* (L.) Urb	Phase 1, double-blind, randomized, crossover clinical trial	Standardized *C.* *asiatica* aqueous extract product (CAP) containing triterpenes and caffeoylquinic acid	10-h observation period after single-dose oral administration (each participant received two doses at least 14 days apart)	4	Patient with Mild Cognitive Impairment	2 g and 4 g (single oral doses)	Antioxidant and neuroprotective effects	[107]
*Crocus* *sativus* L	Multicenter, randomized, double-blind controlled trial	Capsule	22 weeks	54	Patients with mild-to-moderate AD	15 mg twice per day	Improve mild-to-moderate AD	[145]
*Ginkgo* *biloba* L	Phase II, open-label clinical study	*Ginkgo* *biloba* extract (standardized EGb 761	24 weeks treatment	34 enrolled (23 completed 12 weeks, 19 completed 24 weeks)	Brain tumor survivors with radiation-induced cognitive dysfunction	120 mg/day	Improved executive function, attention, concentration, and non-verbal memory; better mood and quality of life	[146]
*Hericium* *erinaceus* Mycelia	Double-blind, randomized, placebo-controlled Study	Capsules	49 weeks	41	Patients with mild AD	350 mg/g three times daily	Decrease cognitive decline Abilities Screening Instrument (CASI), Mini-Mental State Examination (MMSE), and Instrumental Activities of Daily Living (IADL) scores and achieved a better contrast sensitivity in patients with mild AD neurocognitive benefits	[147]
*Huperzia* *Serrata* (Thunb.) Trevis	Double blind study		8 weeks	50	Patients with mild-to-moderate AD	0.2 mg twice a day	Decreasecognitive and task switching deficits in patients with AD	[148]
*Melissa* *officinalis* L	Randomized placebo-controlled double-blind trial	Not reported	96 weeks	323	Patients with mild AD dementia	500 mg of per day	Prevent cognitive decline	[149]
*Nigella* *sativa* L	Randomized placebo-controlled trial	Capsule	9 weeks	40	Memory, attention and cognition in healthy elderly volunteers	500 mg capsules once daily	Increase memory, attention and Cognition. Preventing or Slow progressing of ad	[150]
*Panax* *ginseng* C.A.Mey	Open label study	Powder	12 weeks	97	Patients with mild-to-moderate AD	4.5 g/d	Cognitive subscale of ADAS and the MMSE score began to show improvements, effective in the cognitive performance of AD patients	[151]
*Salvia* *miltiorrhiza* Bunge	Randomized, double-blind, parallel, placebo-controlled, multicenter trial	Tablets	24 week	240	Patients with mild to moderate Vascular dementia (VaD)	0.3 g three times per day	Efficacy and safety for the treatment of patients with VaD	[152]
*Vitis* *vinifera* L	Randomized, double‐blind, placebo‐controlled clinical trial	Dietary supplement Cognigrape^®^	12 weeks	111	Healthy older adults	250 mg/day	Increase attention, memory, and language performance in healthy adults	[153]
Yi-Zhi-An-Shen granules (YZASG) *(Alpinia* *oxyphylla* Miq. *Panax* *notoginseng*, *Ligusticum* *chuanxiong* Hort. *Gardenia* *jasminoides* Ellis, *Curcuma* *longa* L., and *Lophatherum* *gracile)*	Randomized, double-blind, placebo-controlled clinical trial	Yi-Zhi-An-Shen granules	16 weeks	80	Patients with amnestic mild cognitive impairment	Three times a day	Prevent or delay cognitive decline in patients with MCI or dementia	[154]
Polyherbal formulation (*Bacopa* *monnieri* Wettst*,* *Hippophae* *rhamnoides* L. and *Dioscorea* *bulbifera* L.)	Randomized double‐blind placebo‐ and active‐controlled clinical trial	Tablets	12 months	232	Senile dementia of Alzheimer’s type (SDAT) patients	500 mg, twice daily	Increase cognitive functions, decrease inflammation and oxidative stress	[155]
*Panax* *ginseng* C.A.Mey , *Ginkgo* *biloba* L., and *Crocus* *sativus* L	Randomized, placebo‐controlled, double‐blind crossover design pilot study	Not reported	1 week	16	Healthy adults		Increase visuospatial short‐term memory and working memory	[156]
Kami Guibi-tang (KGT) Korean herbal medicine	Randomized, double-blind, placebo-controlled trial	granules	24-week	16	Patients with mild AD	3.0 g with water three times a day	Improves in individuals with mild cognitive impairment	[157]

Notably, the reliability and generalizability of these clinical findings are influenced by considerable variation in sample sizes across studies, ranging from small pilot or phase-1 trials to larger randomized and multicenter investigations. Specifically, the reviewed trials can be broadly categorized into small-sample studies (≤ 40 participants) and large-sample studies (≥ 200 participants), which show clear differences in evidentiary strength. Small-sample investigations, including early-phase or pilot trials, frequently report positive trends in cognitive or biomarker outcomes but are characterized by wide confidence intervals and increased interindividual variability. In contrast, large-scale randomized trials, such as those involving *Melissa* *officinalis* (n = 323), *Salvia* *miltiorrhiza* (n = 240), and polyherbal formulations (n = 232), provide more consistent and statistically robust evidence for cognitive stabilization or improvement, supporting greater reliability and clinical generalizability. While smaller sample sizes are appropriate for early-phase safety, pharmacokinetic, and mechanistic exploration, they may limit statistical power and increase susceptibility to bias or interindividual variability. In contrast, studies with larger cohorts and longer follow-up durations provide more robust evidence for efficacy and clinical relevance, particularly for chronic and progressive conditions such as AD. Therefore, although the collective clinical evidence is encouraging, future trials with adequately powered sample sizes, standardized cognitive endpoints, and multicenter designs are essential to confirm therapeutic efficacy and strengthen the translational potential of medicinal plant-based interventions for AD.

Although this review does not present primary clinical efficacy data, indirect comparisons based on outcomes reported in the existing literature provide useful clinical context. FDA-approved cholinesterase inhibitors such as donepezil are widely reported to confer modest symptomatic cognitive benefits in mild to moderate Alzheimer’s disease, but their use is frequently limited by gastrointestinal, cardiovascular, and sleep-related adverse effects. In contrast, published clinical studies of selected medicinal plants, particularly Crocus sativus and standardized Ginkgo biloba extract (EGb 761^®^), describe cognitive stabilization or modest improvement over comparable treatment durations, often accompanied by improved tolerability and lower incidence of adverse reactions. While heterogeneity in study design, formulation, and outcome measures prevents direct efficacy ranking, these indirect comparisons suggest that certain medicinal plant-based interventions may offer clinically relevant symptomatic benefits with a more favorable safety profile, supporting their role as complementary therapeutic options.

## Safety, adverse effects, and herb–drug interaction of medicinal plants in AD

While many medicinal plants show potential neuroprotective effects in AD, safety and interaction risks must be recognized alongside benefits. Clinical studies indicate that garlic supplementation is generally well tolerated, but adverse effects can occur. In human clinical trials, garlic intake has been associated with gastrointestinal discomfort, nausea, dizziness, headache, and mild orthostatic hypotension, particularly at higher doses, suggesting caution for elderly patients and those with pre-existing conditions. Additionally, because garlic can influence blood fluidity, its use alongside conventional AD therapies that affect vascular or circulatory status should be monitored by clinicians to avoid additive effects on bleeding tendency [158]. *C.* *asiatica* is generally well tolerated in clinical settings, with acute administration of standardized extracts (2–4 g) showing good safety and minimal adverse effects in older adults, including those with cognitive impairment, in phase I pharmacokinetic trials [159]. However, isolated clinical and case reports have documented rare but notable safety concerns: prolonged use has been associated with hepatic dysfunction, including granulomatous hepatitis in individuals taking the herb for weeks to months, suggesting possible hepatotoxicity at higher doses or extended durations [108] and earlier case reports. In vitro enzyme interaction studies indicate that *C. asiatica* extracts exhibit only weak reversible inhibition of major cytochrome P450 isoforms (e.g., CYP2C9) at high concentrations, suggesting a low potential for clinically significant drug metabolism interactions, though in vivo relevance depends on achievable plasma levels of active constituents [160]. For *B.* *monnieri*, clinical intervention studies report it is generally well-tolerated with no serious adverse events, though gastrointestinal discomfort has been noted in some subjects receiving standardized extract (e.g., 300 mg/day) in cognition studies without significant safety concerns over 12 weeks, underscoring mostly mild and transient effects in humans [161]. clinical trials using *G.* *biloba* extract EGb 761 at therapeutic doses (240 mg/day) in patients with dementia and mild cognitive impairment have shown that the extract is generally safe and well tolerated, with no differences in adverse event rates compared with placebo observed across multiple clinical endpoints in larger cohort studies (n≈946 participants across studies) assessing cognitive and neuropsychiatric outcomes in older adults [117]. Standardized *G.* *biloba* extract (EGb 761^®^) has been extensively studied in large randomized clinical trials involving dementia and cognitive impairment. Many studies report that EGb 761^®^ is generally safe and associated with no significant increase in adverse events compared with placebo or conventional therapy such as donepezil. Trials have documented common mild side effects including gastrointestinal symptoms, headache, dizziness, hypertension, and upper respiratory infections in both treatment and control groups [162]. However, safety data from AD trials are limited in scope, and higher doses of saffron (> 1.5 g/day) have been associated with mild toxicity and potential poisoning in broader human research, underscoring dose‑dependent risk. In older adults with impaired renal or hepatic function, such dose considerations are especially important given altered pharmacokinetics [163]. Bacopa’s cholinergic activity, due to bacosides, may interact with cholinergic or muscarinic drugs. Animal research showed additive cholinergic toxicity when combined with the muscarinic agonist cevimeline, manifesting as hyperhidrosis, nausea, dizziness, and tachycardia [164]. Clinical studies of *Melissa* *officinalis* extract containing rosmarinic acid (500 mg daily) in patients with mild Alzheimer’s dementia showed no serious adverse events or safety signals, and no differences in vital signs or neurologic examinations compared to placebo, indicating good tolerability in humans. However, mild gastrointestinal discomfort and rare sedation have been reported in some participants, and theoretical interactions with sedative or cholinergic medications warrant monitoring in AD patients taking concurrent CNS drugs [165]. Although *Nigella* *sativa* (black seed) seed extracts and thymoquinone are generally regarded as having low toxicity in preclinical studies, high doses of certain extracts can induce behavioral changes (sedation/agitation) in animal models, and subchronic exposure may alter liver enzymes at high concentrations, suggesting dose-dependent effects that require cautious dosing in human applications. Limited clinical safety data exist for AD populations specifically, so potential interactions with drugs metabolized via hepatic pathways or with CNS effects should be considered [166]. Direct clinical safety data for *Salvia* *miltiorrhiza* (danshen) in AD are limited. Case reports from other clinical contexts have documented enhanced anticoagulant effects (bleeding risk) when combined with warfarin or antiplatelet drugs, indicating potential interaction concerns in patients on cardiovascular therapies. These interactions are mechanistically linked to effects on platelet aggregation and should be considered in multi-drug regimens common in elderly AD populations.

## Future perspectives and conclusion

Growing evidence presented in this work demonstrates that medicinal plants and their bioactive constituents exert meaningful neuroprotective effects through multi-target mechanisms that align with major pathological features of AD. As shown across the studies summarized, plant-derived compounds regulate oxidative stress and neuroinflammation, modulate amyloid-β and tau pathology, support cholinergic transmission, protect mitochondrial function, enhance synaptic resilience, and influence the gut–brain axis, collectively offering a therapeutic versatility that current single-target drugs lack. These findings underscore the value of natural compounds as promising candidates for next-generation therapeutics; however, their clinical translation requires addressing several critical research gaps. First, despite strong mechanistic evidence, the majority of available data is derived from preclinical models. Although animal and cellular studies demonstrate robust neuroprotective potential, large-scale, well-controlled human clinical trials remain limited. In addition, variability in extract composition, dosing regimens, and outcome measures hampers cross-study comparability. Furthermore, challenges related to poor bioavailability, limited blood–brain barrier (BBB) penetration, and rapid metabolic degradation continue to restrict therapeutic efficacy. These pharmacokinetic hurdles represent major technical bottlenecks in formulation development: many phytochemicals like curcumin, resveratrol, crocin, and bacosides exhibit rapid first-pass metabolism and low aqueous solubility, resulting in low systemic exposure and limited CNS levels despite promising in vitro potency (e.g., curcumin’s oral bioavailability < 1% in humans). To overcome these challenges, formulation science is actively exploring advanced delivery technologies. Nanoparticle-based carriers (e.g., polymeric nanoparticles, solid lipid nanoparticles) have been shown to enhance oral absorption and protect labile phytochemicals from enzymatic degradation, increasing plasma concentrations and CNS exposure in animal models. For example, curcumin encapsulated in phospholipid complexes or liposomal carriers demonstrated significantly higher brain uptake and antioxidant activity versus unformulated curcumin in rodent AD models, suggesting improved BBB penetration and sustained release kinetics. Similarly, phospholipid-based phytosome formulations of Ginkgo biloba and Bacopa monnieri extracts have been shown to increase oral bioavailability and cognitive benefits in early human studies compared with standard extracts. Prodrug strategies and chemical conjugation with carrier peptides or targeting ligands (such as transferrin receptor antibodies) are also under investigation to facilitate receptor-mediated transcytosis across the BBB for compounds with poor innate permeability. To bridge these gaps, future research must prioritize standardized extract preparation, dose optimization, pharmacokinetic and pharmacodynamic profiling, and long-term safety assessment. Given the favorable safety profiles of many phytochemicals, medicinal plants represent viable candidates for preventive interventions, particularly in at-risk populations such as individuals with metabolic syndrome, chronic inflammation, or mild cognitive impairment. Precision phytopharmacology offers another promising solution, whereby compounds are selected based on individual genetic background (e.g., ApoE4 status), metabolic state, inflammatory burden, or microbiome composition. Integrative approaches combining metabolomics, transcriptomics, and microbiome profiling may help identify patient subgroups most likely to benefit from specific botanical interventions. Additionally, synergistic strategies warrant deeper investigation, as multiple plant extracts act on complementary pathways such as Nrf2 activation, cholinergic modulation, and inhibition of amyloidogenesis. Rational design of multi-compound formulations using network pharmacology, computational modeling, and AI-driven screening could substantially accelerate therapeutic development. Increasing evidence also highlights the gut microbiome as a key modulator of AD pathology through immune, metabolic, and neuroendocrine pathways. Many plant-derived compounds, including polyphenols, carotenoids, and alkaloids, favorably influence microbial composition and metabolite production. Future studies should clarify how plant-derived metabolites, short-chain fatty acids, and microbial co-metabolites mediate neuroprotective effects, thereby enabling microbiota-targeted therapeutic strategies. Emerging formulation technologies such as solid lipid nanoparticles, nanoemulsions, cyclodextrin inclusion complexes, and dendrimer-based carriers provide additional platforms to enhance oral and CNS delivery of phytochemicals. Continued optimization of these technologies, combined with safety and scalability assessments, will be essential for clinical translation. Moreover, advances in controlled-release matrices and stimuli-responsive systems (e.g., pH- or enzyme-triggered release) can improve therapeutic windows and reduce dosing frequency, which is particularly important for chronic conditions like AD. In conclusion medicinal plants and their bioactive molecules hold considerable promise as multi-target therapeutic candidates for AD. Across diverse models, these compounds consistently attenuate oxidative stress, suppress neuroinflammation, inhibit amyloid-β aggregation and tau pathology, modulate cholinergic and synaptic function, improve mitochondrial stability, and restore cognitive performance. Such broad-spectrum activity is particularly valuable given the multifactorial and progressive nature of AD. The global increase in AD prevalence, varying widely across regions and shaped by demographic, socioeconomic, and lifestyle determinants, underscores the urgent need for safe, accessible, and population-wide preventive strategies. Plant-based interventions, which have long histories of traditional use and favorable safety profiles, may offer cost-effective solutions that can be integrated into public health frameworks, especially in low-resource settings. Despite the encouraging data, translation into clinical practice requires rigorous, standardized, and mechanistically guided research. Future work should focus on clinical validation, improved delivery systems, personalized approaches, and synergistic formulations. With sustained interdisciplinary efforts, phytochemicals and medicinal plants could form the foundation of novel therapeutic strategies that modify disease progression, support healthy brain aging, and ultimately reduce the global burden of AD.

## Data Availability

The authors nothing to report.

## Abbreviation

Aβ: Amyloid-β
Aβ_1–40_/Aβ_1–42_/Aβ_25–35_: Amyloid-β peptide fragments
ACh: Acetylcholine
AChE: Acetylcholinesterase
ADAS-Cog: Alzheimer’s Disease Assessment Scale-Cognitive Subscale
AD: Alzheimer’s disease
AGE: Aged garlic extract
AKT: Protein kinase B
AlCl_3_: Aluminum chloride
AMPA: α-Amino-3-hydroxy-5-methyl-4-isoxazolepropionic acid receptor
APP: Amyloid precursor protein
BDNF: Brain-derived neurotrophic factor
BChE: Butyrylcholinesterase
BPSDs: Behavioral and psychological symptoms of dementia
BBB: Blood–brain barrier
BCAOO: Bilateral common carotid artery occlusion
Bcl-2: B-cell lymphoma 2
Bax: Bcl-2–associated X protein
CaMKII/p-CaMKII: Calcium/calmodulin-dependent protein kinase II
CAP: *Centella* *asiatica* Aqueous product
CAT: Catalase
CASI: Cognitive abilities screening instrument
CNS: Central nervous system
CREB/p-CREB: CAMP-response-element-binding protein
CZE: Capillary zone electrophoresis
DNA: Deoxyribonucleic acid
ER stress: Endoplasmic reticulum stress
FDA: U.S. Food and Drug Administration
FGAL/F-GAL: Fenugreek galactomannan
G/M ratio: Galactose/mannose ratio
GABA: Gamma-aminobutyric acid
GPx: Glutathione peroxidase
GSH: Reduced glutathione
GSK-3β: Glycogen synthase kinase-3β
H_2_O_2_: Hydrogen peroxide
HT22: Mouse hippocampal neuronal cell line
IA: Incensole acetate
IADL: Instrumental activities of daily living
IFN-γ/IFN-β: Interferon-gamma/interferon-beta
IL-1β/IL-6: Interleukin-1β/interleukin-6
IMR-32: Human neuroblastoma cell line
JNK: C-Jun N-terminal kinase
KGT: Kami Guibi-tang
LDL: Low-density lipoprotein (context from herbal cardiometabolic effects)
LRP1: Low-density lipoprotein receptor-related protein-1
LPS: Lipopolysaccharide
LTP: Long-term potentiation
MAPK/p38-MAPK: Mitogen-activated protein kinase
MCI: Mild cognitive impairment
MCP-1: Monocyte chemoattractant protein-1
MMSE: Mini-mental state examination
MRI: Magnetic resonance imaging (indirect context)
mRNA: Messenger RNA
NADPH: Nicotinamide adenine dinucleotide phosphate
NEU N (NeuN): Neuronal Nuclei marker
NFTs: Neurofibrillary tangles
NF-κB: Nuclear factor kappa-light-chain-enhancer of activated B cells
NMDA: N-methyl-D-aspartate
NO: Nitric oxide
Nrf2: Nuclear factor erythroid 2-related factor 2
P-gp: P-glycoprotein
PC12: Rat adrenal pheochromocytoma cell line
PI3K: Phosphatidylinositol-3-kinase
p-tau: Phosphorylated tau
RAGE: Receptor for advanced glycation end-products
RNA: Ribonucleic acid
ROS: Reactive oxygen species
SAMP10: Senescence-accelerated mouse prone-10
SOD: Superoxide dismutase
SH-SY5Y: Human neuroblastoma cell line
STZ: Streptozotocin
STAT3: Signal transducer and activator of transcription 3
TCM: Traditional Chinese Medicine
TLR4: Toll-like receptor 4
TNF-α: Tumor necrosis factor-alpha
TrkB: Tropomyosin receptor kinase B
VaD: Vascular dementia
WHO: World Health Organization
